# Grass hay mixed-in creep feed or separately-fed differentially affects digestive development in pre- and post-weaning piglets

**DOI:** 10.1186/s40104-025-01227-4

**Published:** 2025-07-01

**Authors:** Renjie Yao, Tetske G. Hulshof, Hubèrt M. J. van Hees, An Cools, Mattijs Merckx, Dominiek Maes, Geert P. J. Janssens

**Affiliations:** 1 Department of Veterinary and Biosciences, Ghent University, Merelbeke, Belgium; 2 Department of Internal Medicine, Reproduction and Population Medicine, Ghent University, Merelbeke, Belgium; 3 Trouw Nutrition Research & Development, Amersfoort, The Netherlands

**Keywords:** Dietary fibre, Gastrointestinal development, Grass hay, Suckling piglets

## Abstract

**Background:**

Based on observations in feral pigs, the role of dietary fibre and structure may be underestimated in suckling piglet nutrition. This study investigated the effect of grass hay offered to suckling piglets either separately or included in their creep feed, combined with nursery diets with or without grass pellet inclusion on growth performance and gastrointestinal development.

**Methods:**

Thirty-six litters (14–15 piglets per litter) were divided into three equal groups of 12 litters per treatment during the suckling phase: control group (CON) received regular creep feed; GH group received chopped grass hay as-is in separate feeders alongside regular creep feed; PGH group received regular creep feed but barley and wheat were replaced by 28% grass pellets. After weaning (d 23), each litter was split into two dietary treatments in a split-plot design (pre-wean treatment as main plot). Two of the pre-wean diets were also offered until d 14 post-weaning, i.e., CON (CON nursery diet, CON-C, GH-C, PGH-C) and PGH (GH nursery diet, CON-GH, GH-GH, PGH-GH). Thereafter, transitioning to a diet containing 13% wheat/barley or grass pellets, respectively, until d 39 post-weaning. Gastrointestinal morphology, gene expression of intestinal nutrient transporters and barrier proteins, metabolite profile and microbiota were assessed on the day before weaning, d 10 and d 38 post-weaning. A total of 24 piglets were sacrificed at each dissection point.

**Results:**

At weaning, GH group had consumed 7 g/piglet grass hay, and PGH group had consumed 46 g/piglet creep feed. One day before weaning, GH piglets showed heavier emptied small intestine (*P* = 0.044) and colon (*P* = 0.065), higher SCFA production in proximal segments and lower SCFA production in colon (*P* < 0.05). Higher abundance of *Prevotellaceae NK3b31 group* was observed in caecal and colonic content of PGH compared to GH group (*P* < 0.05), and PGH group showed a lower energy conversion ratio (net energy intake/gain, *P* = 0.035). Following weaning, GH nursery group had a reduced average daily gain (226 vs. 183 g, *P* < 0.001) during d 0–14, while this group showed compensatory growth afterwards (*P* = 0.056). Main plot effects on increased expressions of *CLDN3* and *FFAR2* were observed in GH and PGH by d 38 post-weaning (*P* < 0.05). An interaction effect showed greater luminal abundance of the *Prevotellaceae NK3b31 group* in GH-GH and PGH-GH groups compared to CON-GH on d 38. The GH nursery diet showed a better energy conversion ratio (*P* = 0.006) with no influence on body weight and their SCFA production shifted towards proximal segments.

**Conclusion:**

In conclusion, feeding a structured and fibre-rich diet to suckling piglets enhance their digestive tract development and adapt their microbiome to fibre digestion in later life. Maintaining a fibre-rich diet from suckling to nursery is recommended, though this come with a transient reduction in weight gain caused by lower feed intake that, however, can be recovered afterwards accompanied with an optimized energy conversion ratio.

**Supplementary Information:**

The online version contains supplementary material available at 10.1186/s40104-025-01227-4.

## Introduction

At the end of the suckling phase, farmed piglets need to abruptly transition from a sow milk-based diet to a solid diet. This requires a resilient gastrointestinal tract (GIT) but developing this continues to pose an issue [1]. In addition to the dietary transition, piglets face complex challenges, including separation from the sow, new housing conditions and stress from newly established social hierarchies. These stressors, combined with the immature physiological conditions in these piglets, result in anorexia, diarrhoea, growth stasis and potentially, mortality [2–5]. Creep feeding—applying a supplemental diet to piglets during the suckling period—is widely applied to accelerate GIT development and familiarize piglets with solid feed prior to weaning. Typically, creep feeds are formulated to be nutrient-dense, highly digestible and palatable. However, creep feed intake remains variable within and among litters, potentially resulting in health and performance problems in later life phases [6, 7]. Meanwhile, excessive fermentation of poorly digested creep feed residues in the distal digestive tract promotes microbial dysbiosis, eventually exacerbating the weaning stress [8].

In contrast to the farm setting, piglets in the wild start ingesting solid, fibre-rich items from the first week of life onward, which is associated with faster development of their stomachs [9]. Studies across diverse wild animal species have reported a drive for particular dietary profiles that are assumed to match their nutritional requirements [10–12]. The observation in wild piglets, therefore, presents promising possibilities for incorporating fibrous and structure-rich items into the diet of suckling piglets. For suckling piglets in farm settings, the precise nutritional support and proper development of digestive organs should be prioritized to support resilience in later phases, rather than solely aiming for maximal growth rate, as is done for the fattening period. Therefore, the previous study reported that replacing starch with insoluble fibre, such as cellulose and oat hulls, in creep feed increased the weight and length of the GIT and subtly altered the microbiome composition in the colon [13, 14]. To further simulate the natural conditions, our previous study demonstrated similar effects in suckling piglets offered chopped grass hay in a separate feeder alongside common creep feed [15]. Grass hay fostered the growth of the GIT and was well-accepted by suckling piglets. The variable physical and chemical properties of different sources of dietary fibre, however, may result in distinct modes of action [16]. Meanwhile, whether the more robust GIT development observed at weaning in the previous study can be maximally utilized or enhanced by continuing grass hay inclusion in the post-weaning diet, remains to be clarified [15]. Thus, further research is warranted to optimize the provision of dietary fibre and structure both in the pre- and post-weaning phases.

Given that finely milled and pelleted diets are commonly applied in practice and postulating that some outcomes in our previous study may be attributed to limited sample size, this current study not only replicated the chopped grass hay treatment on a larger-scale, but also used a pelleted grass-containing diet for suckling piglets. Additionally, this study aimed to explore the interactive effect of feeding grass hay during the suckling and nursery phase on growth performance, gastrointestinal development, gene expression of nutrient transporters and intestinal barrier proteins, gastrointestinal metabolites, and microbiota. We hypothesized that a long-term, fibre-enriched diet initiated at a young age would further benefit the overall development of piglets.

## Materials and methods

The housing, rearing and any other procedures on animals were in compliance with the European Union Directive 2010/63/EU, and assessed by the Dutch Central Committee on Animal Experimentation (CCD), under application number AVD20400202316684.

### Animal housing and management

Thirty-six Hypor Libra sows (Hendrix Genetics, Boxmeer, The Netherlands; average parity 3.16, ranging from 1 to 6,) were inseminated (Hypor Maxter) at the Swine Research Centre (Trouw Nutrition R&D, Sint Anthonis, The Netherlands). They were moved to individual farrowing pens (200 cm × 260 cm) one week prior to the expected farrowing date. Ten farrowing pens were located in each climate-controlled room, where the lights were on between 6:00 and 22:00. Each pen was equipped with separate drinking nipples for the sow and the piglets, and an elevated feed trough for the sow designed to prevent piglets from consuming sow feed. Sows had ad libitum access to drinking water and were fed daily rations according to a step-up scheme after farrowing. The day when most sows started farrowing was set as d 0 of this trial, and litter size was equalized over treatments to 14–15 piglets per sow within 2 d after farrowing. Ear tagging, tail docking and iron injection were performed within 3 d after birth of piglets. All piglets were vaccinated against *Escherichia coli* serotype F18 (Ecoporc Shiga, IDT Biologika GmbH, Dessau-Rosslau, Germany) on d 3 of age, and a triple-vaccine against *Mycoplasma hyopneumoniae,* porcine circovirus type 2 (PCV2) and porcine reproductive and respiratory syndrome (PRRS) virus (Ingelvac Mycoflex, Circoflex and PRRSflex, respectively, Boehringer Ingelheim GmbH, Ingelheim, Germany) was administered 1 week prior to weaning. Weaning occurred on a fixed day, with piglets averaging 23 d of age (SD = 0.8 d) and 6.6 kg body weight (SD = 0.5 kg).

### Experimental design and dietary treatments

**Fig. 1:**
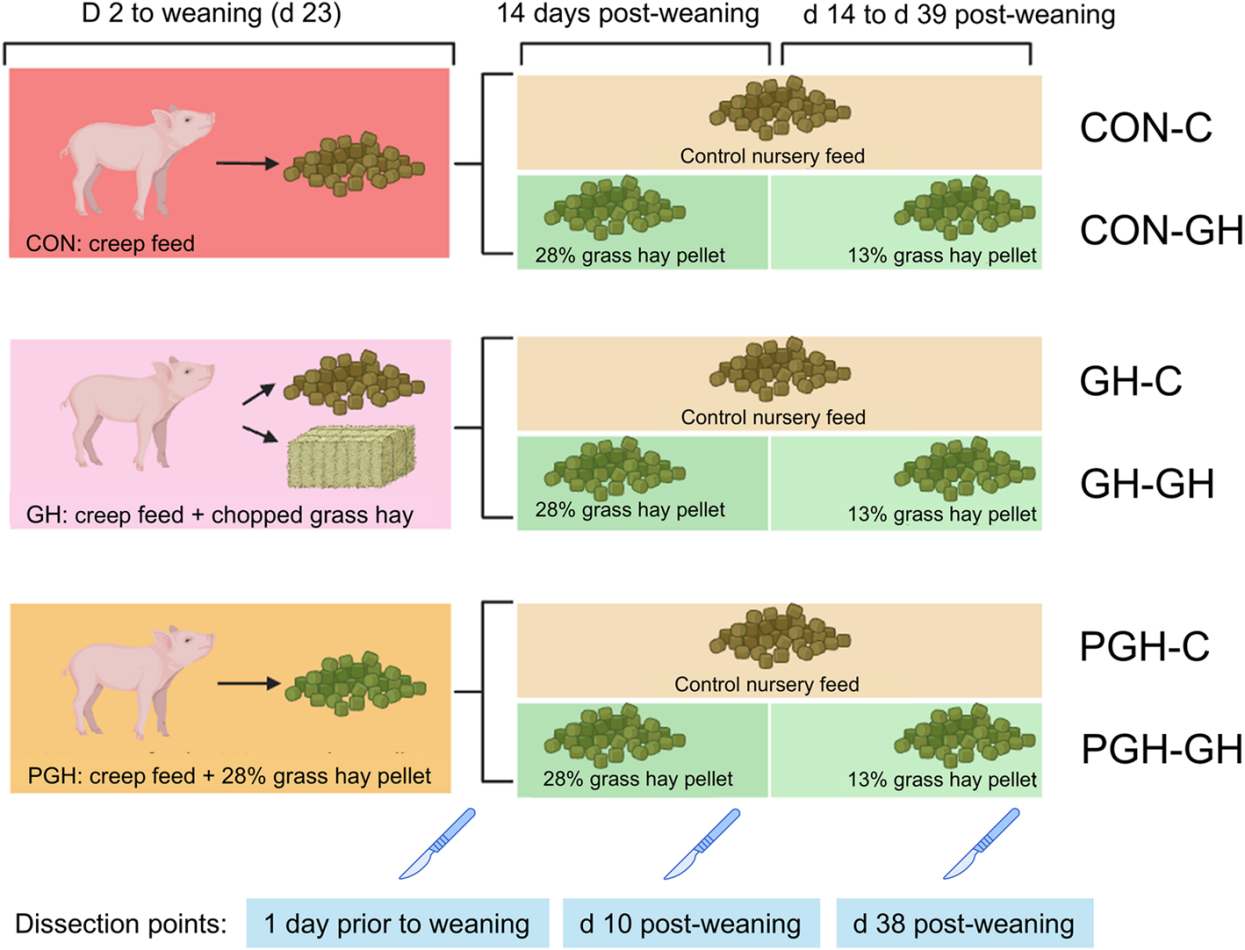
The schematic diagram from the start of the treatments (d 2 of age) to the end of the experiment (d 39 post-weaning)

This study was designed as split-plot with 3 dietary treatments pre-weaning as main plot (experimental unit was litter) and 2 dietary treatment post-weaning as split plot (experimental unit was pen; Fig. 1). Pre-weaning, litters were randomly allocated to a treatment based on parity and farrowing date of the sow. From d 2 of age until weaning, the piglets in the control group (CON, *n* = 12 litters) only received the control creep feed which consisted of a basal diet containing highly digestible, finely ground ingredients and 28% of a 50:50 mix of wheat and barley in a round feeder (Table 1). In addition to the control creep feed, piglets in the GH group (*n* = 12 litters) were given chopped grass hay (analysed nutrients: 8.7% crude protein, 1.5% crude fat, 59.35% neutral detergent fibre and 32.4% acid detergent fibre) in a separate feeder alongside the creep feed feeder. The positions of the two feeders were alternated daily to avoid location preference bias in pens of the GH group. The third group (PGH, *n* = 12 litters) received the control diet in which the 28% wheat and barley were fully replaced by a grass pellet (mean particle size: 0.36 mm). The diets were fed as crumble pre-wean and for the first 14 d post-wean and their composition can be found in Table 1. The inclusion level of the grass pellet was selected based on the result of voluntary feed intake observed in a previous study [15]. The feeders in the pen were positioned identically across all litters and near the head of the sow. Creep feed, grass hay and water were available ad libitum for piglets.

**Table 1:** Composition (ingredients, nutrients) of the experimental creep feeds fed from d 2 to weaning

Items	CON^1^	PGH^2^ (28% grass pellet)
Ingredient composition, g/kg as-fed basis		
Wheat	140	-
Barley	140	-
Basal diet creep feed^9^	700	700
Soybean oil	20	20
Grass pellet	-	280
Total	1,000	1,000
Analysed nutrient composition, g/kg as-fed basis		
Moisture	97	87
Crude protein	174	166
Crude fat	67	64
Ash	46	65
Soluble dietary fibre	44	39
Insoluble dietary fibre	128	259
NDF^3^	107	230
ADF^4^	35	117
ADL^5^	8	21
Calculated nutrient composition, per kg as-fed basis^10^		
ME^6^, kcal	3,404	3,012
NE^7^, kcal	2,519	2,169
SID^8^ Lys, g	11.8	11.6
SID Met + Cys, g	7.5	6.9
SID Thr, g	7.7	7.5

^1^CON =  Control group; ^2^PGH = Grass pellet group

^3^NDF = neutral detergent fibre; ^4^ADF = acid detergent fibre; ^5^ADL = acid detergent lignin

^6^ME = metabolic energy; ^7^NE = net energy; ^8^SID = standardized ileal digestibility

^9 ^Basal diet creep feed consisted of wheat (13%); barley (15%); extruded cereals (29%); soybean products (16%), including extruded soybean meal (Forcital, Trouw Nutrition, Ghent, Belgium); dairy whey products (8.5%); fats and oils (4.2%); vitamin and minerals premix (6.1%); sucrose and palatability enhancers (3.2%); wheat protein (1.9%); potato protein (2.6%); synthetic amino acids (0.8%) and organic acids as feed preservatives (0.1%)

^10 ^Composition was calculated according to NRC 2012 standards

Feed leftovers were collected and completely replaced by fresh feed at 7:00 each day. Concurrently, feeders were replenished with fresh feed when empty which was checked twice daily during the first two days, and subsequently four times daily. Three days prior to weaning, piglets were identified as creep feed eaters by adding a blue colorant (0.5% indigo carmine from Sigma-Aldrich) to the crumbles for faecal colour assessment from d 8 to 4 prior to weaning. Two days prior to weaning, 8 litters per treatment (out of the 12 in total) were selected based on number of eaters with 4 litters per treatment having at least 1 eater and 4 other litters per treatment having at least 5 eaters.

Following weaning, piglets were moved to a nursery unit. The 12 piglets closest to median weight of each litter was split into two equal groups of 6 piglets and housed in a pen (dimensions 200 cm × 150 cm), ensuring a balanced body weight distribution between the two pens per litter. The piglets selected for post-wean sampling were evenly distributed in two nursery pens. One pen from each litter received the control feed (CON nursery group: CON-C, GH-C PGH-C), while the other pen received the diet with 28% grass pellet (GH nursery group: CON-GH, GH-GH, PGH-GH) during the first 14 d post-weaning. These diets were the same as used pre-weaning. On d 14 post-weaning, both groups transitioned to a link diet which consisted of a basal link diet to which either 13% of a 50:50 mix of wheat and barley was added (CON nursery group) or 13% grass pellet (GH nursery group, Table 2). Diets were fed as 4 mm pellets until the end of the study (d 39 post-weaning).

**Table 2:** Composition (ingredients and nutrients) of the experimental nursery feed fed from d 14 to 39 post-weaning

Items	CON nursery diet	GH nursery diet (13% grass pellet)
Ingredient composition, g/kg as-fed basis		
Wheat	65	-
Barley	65	-
Basal diet link feed^1^	850	850
Soybean oil	20	20
Grass pellet	-	130
Total	1,000	1,000
Analysed nutrient composition, g/kg as-fed basis		
Moisture	104	105
Crude protein	163	161
Crude fat	61	62
Ash	52	59
Soluble dietary fibre	41	30
Insoluble dietary fibre	162	218
NDF	145	198
ADF	49	87
ADL	8	14
Calculated nutrient composition, per kg as-fed basis^2^		
ME, kcal	3,272	3,089
NE, kcal	2,370	2,208
SID Lys, g	10.9	10.8
SID Met + Cys, g	6.6	6.3
SID Thr, g	7.7	7.6

*NDF* Neutral detergent fibre, *ADF* Acid detergent fibre, *ADL* Acid detergent lignin, *ME* Metabolic energy, *NE* Net energy, *SID* Standardized ileal digestibility

^1 ^Basal diet link feed consisted of wheat (36%); barley (34%); soybean products (19%), including extruded soybean meal (Forcital, Trouw Nutrition, Gent, Belgium); chicory pulp (3.5%); fats and oils (1.2%); vitamin and minerals (4.6%); palatability enhancers (0.2%); synthetic amino acids (1.3%)

^2 ^Composition was calculated according to NRC 2012 standards

### Sampling and measurement

The individual body weight of piglets was measured at birth, 24 h of age (to calculate colostrum intake), d 10 during lactation (with d 0 being the start of the treatments), the day prior to weaning, d 14 post-weaning, and the last day of the study (d 39 post-weaning). Average daily gain (ADG, g/pig/d) was calculated at litter or pen level per period (d 0 to weaning, weaning to d 14 post-weaning and d 14 to 39 post-weaning). Feed intake during suckling was measured daily and total feed intake of creep feed (g/piglet) and GH intake was calculated (g/litter). During the nursery phase, feed intake was measured on d 14 and 39 and average daily feed intake (ADFI, g/pig/d) was calculated. Feed conversion ratio and energy (FCR) conversion ratio (ECR) were also calculated. Upon discovering a deceased piglet, the animal was weighed, and feed was weighed back to adjust feed intake records accordingly.

Three dissection points were chosen: the day before weaning to evaluate the effect of treatments during lactation and d 10 and d 38 post-weaning. Piglets selected for euthanasia and sampling had similar colostrum intake and a median body weight on d 10 prior to weaning. Thus, on 2 d prior to weaning, all piglets subjected to sampling were selected. Eight piglets per treatment were sacrificed on the day prior to weaning, and 4 piglets per pre-wean * post-wean treatment combination were sacrificed on d 10 and d 38 (*n* = 24 in total per dissection point).

Before dissection, piglets were sedated using a mixture of Zoletil (250 mg zolazepam and 250 mg tiletamine; VIRBAC, Carros, France) and 20 mL Sedanum (20 mg xylazine/mL; Dechra Pharmaceuticals, Northwich, UK) at 1 mL per 10 kg BW and subsequently killed via intra-cardiac injection with 40% barbiturate pentobarbital (390 mg pentobarbital sodium and 50 mg phenytoin sodium per mL, Euthasol, Virbac, Carros, France). A midline laparotomy was performed to expose internal organs. Different sections of the GIT were identified, and clamps were used to avoid mixing of luminal content. The full weights of stomach, small intestine, cecum and colon were measured, their content was sampled for further analysis by gently squeezing, and subsequently their empty weights were recorded. The length of the small intestine and colon was also measured. Additionally, approximately 0.5 cm^2^ tissue samples were collected at consistent positions from the middle of the ileum and colon and preserved in RNAlater solution (R0901, Sigma-Aldrich, USA) at 4 °C for subsequent gene expression analysis. Representative and homogenized digesta from stomach, ileum, cecum and mid-colon were collected. A subsample of the cecum and mid-colon content was collected in a cryo tube and stored at −80 °C for analyses of microbiota. The remaining content samples were stored at −20 °C until further analysis.

### Diet composition analysis

The crude protein, fat, fibre in the feed were analysed by proximate analysis [17]. The combustion method (method 990.03; LECO FP 528 MI, USA) was used to determine the level of nitrogen with protein calculated as N × 6.25. Fat was extracted from the feed using diethyl ether acid hydrolysis. A feed sample was combusted at a temperature of 550 °C after which the residue was weighed to determine ash content [18]. The analysis of cellulose, hemicellulose and lignin was based on the previous methods described in the literature [19].

### Luminal contents’ metabolic profile

The short-chain fatty acids (SCFA) in digesta were analysed using a gas-chromatographic method which is described by Gadeyne et al. [20]. In short, a homogenous 1 g sample was diluted in 5 mL distilled water with internal standard (1 mg of 2-ethyl butanoic acid). After 15 min of centrifugation (22,000 × *g* at 4 °C), the supernatant was filtered, and an aliquot was transferred into a 1.5-mL glass vial for analysis. The SCFA concentrations were measured by gas chromatography (HP 7890 A, Agilent Technologies, Diegem, Belgium), equipped with a flame ionization detector and a Supelco Nukol capillary column (30 m × 0.25 mm × 0.25 μm, Sigma-Aldrich, Diegem, Belgium).

Ammonia-N (nitrogen as NH_4_^+^ and NH_3_) was analysed using colorimetry using the Berthelot reaction as described by Chaney and Marbach [21].

### RNA extraction and quantitative real-time PCR (qRT-PCR)

The gene expressions of *SGLT1*, *B0AT1* and *PepT1* in the ileum, and *ZO-1*, *CLDN3*, *FFAR2* and *MCT1* in the colon were determined by qRT-PCR. The messenger RNA (mRNA) of samples were entirely extracted using TRIzol reagent (Sigma-Aldrich, Overijse, Belgium) in accordance with the manufacturer’s protocol. After examination of the concentration and quality of RNA, the complementary DNA (cDNA) was synthesized from 200 ng of total RNA using the PrimeScript™ RT Reagent Kit (RR037 A, Takara; Saint-Germain-en-Laye, France). mRNA expression was performed in the Lightcycler 480 II detection system (Roche) with Fast SYBR Green Master Mix (Takara). All samples were run in triplicate. The genes hydroxymethylbilane synthase (*HMBS*), succinate dehydrogenase complex subunit A (*SDHA*), tyrosine 3-monooxygenase/tryptophan 5-monooxygenase activation protein zeta (*YWHAZ*), topoisomerase II beta (*TOP2B*), beta-actin (*β-actin*) and TATA-box binding protein (*TBP*) were all used as endogenous control. The overview of primers is listed in Additional file 1: Table S1. Subsequently, the 2^−ΔΔCt^ method was used to analyse the relative fold changes.

### Microbiota analysis in cecum and mid-colon based on 16S rRNA

The microbial DNA in samples were extracted using QIAamp DNA Fecal Mini Kit (Qiagen, Hilden, Germany). DNA samples with concentrations of 1 ng/μL were sent out to Novogene (Beijing, China), where the 16S rRNA gene V3–V4 hypervariable region was amplified. With 15 µL of Phusion^®^ High-Fidelity PCR Master Mix (New England Biolabs), the react conditions were carried out for 0.2 µmol/L of forward and reverse primers, and about 10 ng template DNA. Thermal cycling consisted of initial denaturation at 98 °C for 1 min, followed by 30 cycles of denaturation at 98 °C for 10 s, annealing at 50 °C for 30 s, and elongation at 72 °C for 30 s and 72 °C for 5 min. The Illumina libraries were pooled and size-selected by preparative gel electrophoresis. Sequencing was performed on the Illumina NovaSeq platform (Beijing, China). Raw sequences from all samples underwent filtering, denoising, merging, and chimera removal to form operational taxonomic units (OTU) by the DADA2 plugin in Qiime2 software.

### Statistical analysis

A linear mixed model in SPSS version 29.0 software (IBM SPSS Inc., USA) was used to determine the statistical difference, using litter in the suckling phase, pen during the post-weaning phase, and piglet for post-mortem measurements as the experimental unit. For measurements during the suckling period including post-mortem measurements, sow parity and litter were included as random factors and suckling treatment as fixed factor. Average birth weight after cross-fostering, exact weaning age for performance data during the suckling phase, and body weight at dissection for GIT macroscopy measurements were included as covariates. For measurements during the nursery period, suckling treatment, post-weaning treatment, and their interaction were included as fixed effects and suckling treatment nested in litter was included as random factor. Feed conversion ratio was calculated as ADFI divided by ADG and energy conversion ratio was calculated as (net energy level of the diet × ADFI) divided by ADG. The data of metabolic profiles that was below the detection limit were considered missing values. The Tukey–Kramer correction was applied for multiple comparisons of estimated marginal means. The abundance results at the genus level of microbiota were compared by Kruskal–Wallis H test with Welch’s post-hoc test. A principal coordinates analysis (PCoA) of OTUs based on Unweighted UniFrac distances combined with ANOSIM test. *P* < 0.05 was considered significant and 0.05 ≤ *P* < 0.10 was interpreted as a trend.

## Results

### Performance metrics

Birth weight was 10% lower in the GH group than in the two other pre-weaning groups (*P* = 0.039; Table 3). Before weaning, no statistical difference was observed in creep feed intake among groups (Table 3). Litters from the GH group consumed 7.3 g/piglet chopped grass hay during the complete lactation period (21 d). Even though ADG (*P* = 0.389) was not influenced by suckling treatments, the ECR was significantly improved in the PGH group compared to CON (*P* = 0.035).

**Table 3:** Growth performance of suckling piglets fed control creep feed (CON), control creep feed and chopped grass hay (GH), or creep feed containing 28% grass pellets (PGH)

Items	CON	GH	PGH	SEM^3^	*P*_diet_
Number of litters	12	12	12	-	-
Average sow parity	3.2	3.2	3.2	0.5	1.000
Birth weight, kg	1.47^a^	1.33^b^	1.51^a^	0.06	0.039
Weaning weight	6.7	6.5	6.6	0.2	0.719
Colostrum intake, g/piglet	448	423	468	21	0.351
Total creep feed intake, g/piglet^4^	60.8	53.1	46.8	12	0.675
Total grass hay intake, g/piglet	-	7.3	-	-	-
ADG during lactation, g/piglet/d^4^	226	229	219	8.4	0.389
FCR, g/kg^1^	0.011	0.011	0.009	0.003	0.808
ECR, kcal/g^2^	0.026^b^	0.018^ab^	0.014^a^	0.004	0.035

^1^ FCR = feed conversion ratio: feed intake per day (g)/daily weight gain (kg)

^2^ ECR = energy conversion ratio: the net energy consumed by piglets (kcal)/the weight gain of piglets (g)

^3^ Pooled standard error of the mean

^4 ^The birth weight and the colostrum intake were used as a covariate

^a,b^ Values within a row with different superscripts differ significantly at *P* < 0.05

After weaning, five piglets died and subsequently removed (1 in CON-C, 1 in GH-C, 1 in GH-GH and 2 in PGH-C). A tendency for an interactive effect was observed for body weight at d 14, with only PGH-GH showing a tendency of higher weight compared to the other suckling treatments within GH nursery group (*P* = 0.084; Table 4). The GH nursery group showed significant reduction in growth performance, with lower ADFI (223 vs. 261 g, *P* = 0.001) and ADG (181 vs. 226 g, *P* < 0.001) compared to those in the CON nursery group (Table 4). However, the ECR was lower in the GH nursery group (*P* = 0.001). When the grass pellet inclusion was switched from 28% to 13% at d14 post-weaning, the ADFI and ADG in the GH nursery group increased and exceeded those in the CON nursery group (*P* = 0.002 and *P* = 0.056, respectively), with the ECR remaining lower and increasing FCR compared to the CON nursery group (*P* = 0.006 and *P* = 0.001; Table 4).

**Table 4:** Growth performance of piglets fed with a control diet (CON nursery treatment) or a diet containing 28% (phase 1; d 0–14) or 13% (phase 2; d 14–39) grass pellets (GH nursery treatment) during the post-weaning period after being fed a control creep feed (CON), control creep feed and chopped grass hay (GH), or creep feed containing 28% grass pellets (PGH) during the suckling phase

Nursery treatments	CON			GH			SEM^5^	*P*_sucklin__g_	*P*_nursery_	*P*_suckling×nursery_
Suckling treatments	CON	GH	PGH	CON	GH	PGH				
Number of pens	12	12	12	12	12	12	-	-	-	-
Weaning weight, kg	6.72	6.63	6.69	6.72	6.55	6.80	0.17	0.607	0.956	0.875
Phase 1 (d 0–14)										
Weight at d 14, kg	10.1*	10.0*	10.1*	9.43	9.04	9.74	0.25	0.483	**0.001**	0.084
ADFI^1^, g/d/piglet^6^	261*	261*	263*	221	216	234	13	0.912	**0.001**	0.773
ADG^2^, g/d/piglet^6^	225*	226*	228*	182	168	194	11	0.553	** < 0.001**	0.240
FCR^3^	1.16	1.16	1.16	1.22*	1.22*	1.20*	0.022	0.897	**0.001**	0.954
ECR^4^	2.76*	2.75*	2.73*	2.66	2.65	2.60	0.02	0.902	**0.001**	0.967
Phase 2 (d 14–39)										
Weight at d 39, kg	23.0	22.5	23.0	22.4	21.2	23.3	0.6	0.223	0.424	0.635
ADFI, g/d/piglet^6^	705	688	709	763*	754*	814*	17	0.099	**0.002**	0.188
ADG, g/d/piglet^6^	514	490	507	519*	504*	547*	13	0.149	0.056	0.501
FCR, g/g	1.39	1.41	1.41	1.45*	1.45*	1.48*	0.02	0.451	**0.001**	0.453
ECR, kcal/g	3.29*	3.35*	3.35*	3.20	3.21	3.26	0.06	0.425	**0.006**	0.827

^1 ^ADFI = average daily feed intake in g/pig/d; ^2^ADG = average daily gain in g/pig/d

^3^ FCR = ADFI/ADG

^4^ ECR = (NE of the diet × ADFI)/ADG

^5^ Pooled standard error of the mean

^6^ The initial weight at the beginning of each phase was used as a covariate

^*^ Indicates the significantly increased values within a row based on nursery treatments at *P* < 0.05

### Gastrointestinal morphometrics

On the day prior to weaning, the inclusion of chopped GH increased the empty small intestine weight to 222 g and length to 851 cm (*P* = 0.044 and *P* = 0.012, respectively) compared to CON (214 g and 792 cm, respectively) and PGH (202 g and 758 cm, respectively). Meanwhile, a trend towards a heavier empty colon in GH piglets was observed (*P* = 0.065, Table 5).

**Table 5:** Gastrointestinal tract morphometrics of piglets euthanized on the day prior to weaning^1^

Items^4^	CON	GH	PGH	SEM^3^	*P*_diet_
Emptied stomach weight, g	40	39	40	2	0.840
Full SI^2^ weight, g	227^b^	240^a^	220^b^	4	0.033
Emptied SI weight, g	214^b^	222^a^	202^b^	5	0.044
Length of SI, cm	792^b^	851^a^	758^b^	17	0.012
Full caecum weight, g	27	25	25	3	0.847
Emptied caecum weight, g	12	11	12	1	0.690
Full colon weight, g	75	72	69	6	0.739
Emptied colon weight, g	46	50	44	2	0.065
Length of colon, cm	117	128	125	3	0.074

^1 ^Piglets were fed one of three experimental diets: control creep feed (CON), control creep feed and chopped grass hay (GH), or creep feed containing 28% grass pellets (PGH)

^2 ^SI = small intestine

^3^ Pooled standard error of the mean

^4^ The weight at dissection was used as a covariate

^a,b^ Values within a row with different superscripts differ significantly at *P* < 0.05

At d 10 post-weaning, heavier full and empty colon weights were observed in the GH nursery group (*P* = 0.015 and *P* = 0.048, respectively). Greater colon length was also found in the GH nursery group, averaging 170.7 cm compared to 155.3 cm in the CON nursery group (*P* = 0.043; Table 6). At d 38, the empty weights of stomach (*P* = 0.088), small intestine (*P* = 0.099), and colon (*P* = 0.010) were 17%, 8% and 24% greater, respectively, in the GH nursery group compared to the CON nursery group. However, the SI was shorter in the GH suckling group compared to other two suckling treatments on d 38 post-weaning (*P* = 0.026; Table 6).

**Table 6:** Gastrointestinal tract morphometrics of piglets euthanized at post-weaning d 10 or d 38^1^

Nursery treatments	CON			GH			SEM^3^	*P*_suckling_	*P*_nursery_	*P*_suckling×nursery_
Suckling treatments	CON	GH	PGH	CON	GH	PGH				
Necropsy at d 10 post-weaning^4^										
Emptied stomach weight, g	63	57	59	62	58	61	2	0.031	0.535	0.956
Full SI^2^ weight, g	504	499	486	528	530	549	42	0.916	0.208	0.652
Emptied SI weight, g	391	372	372	369	364	380	27	0.892	0.959	0.968
Length of SI, cm	1,057	1,033	1,076	1,015	1,006	908	48	0.202	0.175	0.507
Full cecum weight, g	54	60	47	68	72	56	7	0.383	0.248	0.507
Emptied cecum weight, g	18	18	17	19	20	18	2	0.780	0.558	0.975
Full colon weight, g	292	274	277	441*	350*	411*	33	0.428	**0.015**	0.772
Emptied colon weight, g	138	116	117	160*	125*	160*	10	0.226	**0.048**	0.232
Length of colon, cm	155	155	156	172*	175*	165*	7	0.315	**0.043**	0.716
Necropsy at d 38 post-weaning^4^										
Emptied stomach weight, g	141	147	149	184*	158*	165*	9	0.470	0.088	0.145
Full SI weight, g	1,257	1,304	1,411	1,597*	1,466*	1,485*	99	0.397	0.124	0.683
Emptied SI weight, g	863	897	872	968*	945*	951*	39	0.974	**0.049**	0.764
Length of SI, cm	1,308^a^	1,223^b^	1,354^a^	1,269^a^	1,159^b^	1,288^a^	64	**0.026**	0.839	0.958
Full cecum weight, g	233	311	182	243	287	290	37	0.560	0.768	0.417
Emptied cecum weight, g	68	66	60	56	65	65	4	0.278	0.946	0.617
Full colon weight, g	1,017	1,015	779	1,298	1,151	1,199	150	0.253	0.114	0.092
Emptied colon weight, g	416	409	404	529*	497*	497*	27	0.688	**0.010**	0.452
Length of colon, cm	250	263	253	289	274	265	14	0.919	0.768	0.837

^1 ^Piglets were fed a control diet (CON nursery treatment) or a diet containing 28% (phase 1; d 0–14) or 13% (phase 2; d 14–39) grass pellets (GH nursery treatment) during the post-weaning period after being fed a control creep feed (CON), control creep feed and chopped grass hay (GH), or creep feed containing 28% grass pellets (PGH) during the suckling phase

^2^ SI = small intestine

^3^ Pooled standard error of the mean

^4^ The weight at slaughter was used as a covariate

^a,b^ Values within a row with different superscripts differ significantly based on suckling treatments at *P* < 0.05. Interaction effects were primarily marked, when the significant difference of overall mean of each suckling treatment was also observed

^*^ Indicates the significantly increased values within a row based on nursery treatments at *P* < 0.05

### The mRNA expression of intestinal nutrient transporters and barrier function in ileum and mid-colon

**Fig. 2:**
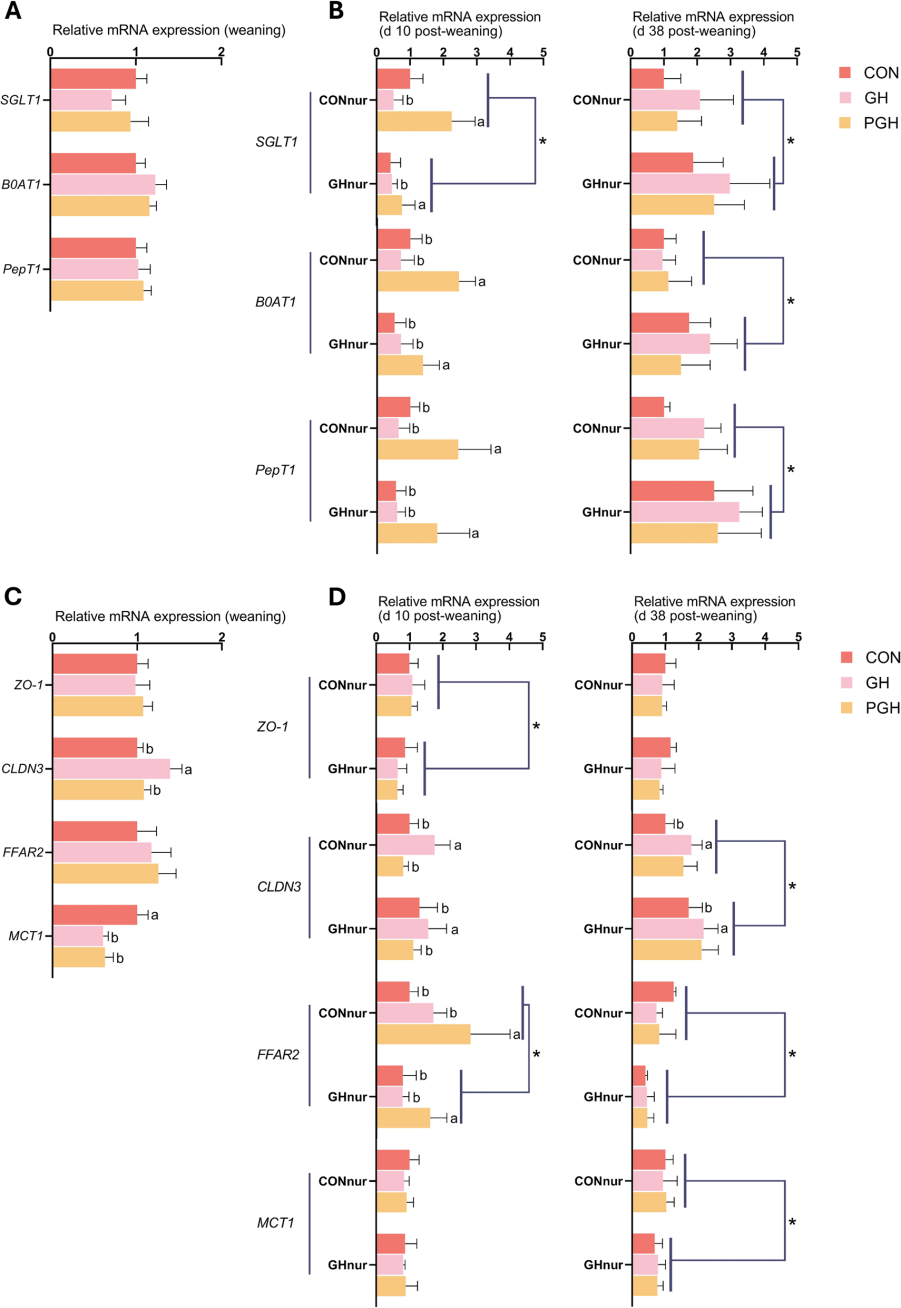
The mRNA expression of nutrient transporters, tight-junction proteins and receptors in ileum and mid-colon of piglets fed a control diet (CONnur) or a diet containing 28% (phase 1; d 0–14) or 13% (phase 2; d 14–39) grass pellets (GHnur) during the post-weaning period after being fed a control creep feed (CON), control creep feed and chopped grass hay (GH), or creep feed containing 28% grass pellets (PGH) during the suckling phase. **A **and** B** The mRNA expression of *SGLT1*, *BOAT1* and *PepT1* in the ileum at weaning and post weaning. **C **and** D** The mRNA expression of *ZO-1*, *CLDN3*, *FFAR2* and *MCT1* in the mid-colon at weaning and post weaning. Bars in the graph represent means ± SD (with each bar based on *n* = 8 at weaning and *n* = 4 post weaning). *SGLT1* = sodium/glucose cotransporter 1; *B0AT1* = Sodium-dependent neutral amino acid transporter B(0)AT1; *PepT1* = Peptide transporter 1; *ZO-1* = Tight junction protein ZO-1; *CLDN3* = Claudin 3; *FFAR2* = Free fatty acid receptor 2; *MCT1* = Monocarboxylate transporter 1. ^a,b^Bars with different superscripts within nursery treatments differ significantly based on suckling treatments at *P* < 0.05; *Indicates a significant difference within a protein based on nursery treatments at *P* < 0.05

In the ileum at the day before weaning, no statistical differences were observed in expression levels of *SGLT1*, *B0AT1* and *PepT1* (Fig. 2A). By d 10 post-weaning, piglets from the PGH suckling group showed higher expression of all evaluated genes compared to piglets from other suckling treatments within each nursery treatment (*P* < 0.05; Fig. 2B). Additionally, the *SGLT1* in the CON nursery group showed higher expression level than in the GH nursery group (*P* = 0.041). At d 38 post-weaning, no significant differences were based on suckling treatments, but all three transporter genes exhibited higher expression levels in the GH nursery group compared to the CON nursery group (*P* < 0.05).

The *CLDN3* expression in the colon was higher in piglets from the GH suckling group than the other two suckling treatments and this effect lasted throughout the study (Fig. 2C and D, *P* < 0.05). Relative to the baseline set as CON, *MCT1* in the colon of piglets from both the GH and PGH suckling groups showed a lower expression on the day prior to weaning (*P* = 0.024; Fig. 2C). Greater expression of *FFAR2* was observed in piglets from the PGH suckling group irrespective of nursery treatment at d 10 post-weaning. For nursery treatments, *ZO-1* and *FFAR2* expression levels at d 10 post-weaning, and *FFAR2* and *MCT1* at d 38 post-weaning showed greater expression in the CON nursery group, while CLDN3 expression was higher in the GH nursery group at d 38 post-weaning (*P* < 0.05; Fig. 2D).

### Metabolic profiles

At the day prior to weaning, the GH suckling group had higher concentrations of acetate (*P* = 0.021) and propionate (*P* = 0.047) in cecum compared to CON, while ammonia concentration (*P* = 0.009) was lower than CON with PGH being in between and not different. The colonic acetate was higher in CON compared to the other two groups (175 vs. 140 vs. 141 µmol/g DM, *P* = 0.018; Table 7).

**Table 7:** Metabolic profiles in gastrointestinal sections of piglets euthanized on the day prior to weaning^1^

Items	CON	GH	PGH	SEM^2^	*P*_diet_
Ileal content at weaning					
Acetate, µmol/g DM^3^	217	257	220	26	0.247
Propionate, µmol/g DM	5.82	7.49	5.30	0.99	0.093
Ammonia, mg/g DM	55	68	73	9	0.403
Caecal content at weaning					
Acetate, µmol/g DM	509^b^	558^a^	521^ab^	8	**0.021**
Propionate, µmol/g DM	185^b^	209^a^	195^ab^	12	**0.047**
Butyrate, µmol/g DM	75	91	83	7	0.085
Valerate, µmol/g DM	31	26	29	6	0.713
BCFA^4^, µmol/g DM	53	44	41	5	0.088
Ammonia, mg/g DM	5.69^a^	4.22^b^	4.84^ab^	0.42	**0.009**
Colonic content at weaning					
Acetate, µmol/g DM	175^a^	140^b^	141^b^	19	**0.018**
Propionate, µmol/g DM	50	37	39	6	0.113
Butyrate, µmol/g DM	17	13	15	3	0.309
Valerate, µmol/g DM	6.95	5.00	5.60	2.61	0.747
BCFA, µmol/g DM	14	9.90	9.60	1.14	0.086
Ammonia, mg/g DM	2.87	2.92	3.09	0.22	0.427

^1^Piglets were fed one of three experimental diets: control creep feed (CON), control creep feed and chopped grass hay (GH), or creep feed containing 28% grass pellets (PGH)

^2^ Pooled standard error of the mean

^3^ DM = dry matter

^4^ Sum of iso-butyrate and iso-valerate

^a,b^Values within a row with different superscripts differ significantly based on suckling treatments at *P* < 0.05

At d 10 post-weaning, the caecal acetate and propionate of the GH suckling group still exhibited higher concentrations than those from the other two (*P* < 0.05), but this effect was only observed within the CON nursery group (interaction *P* = 0.037). An interactive effect was observed, with higher colonic acetate levels in piglets from PGH-GH compared to GH-GH. However, both GH-C and PGH-C showed higher acetate levels than CON-C (suckling *P* < 0.05; interaction *P* = 0.062). Nursery treatment effects were evident at d 10 post-weaning, with higher concentrations of caecal propionate, butyrate and ammonia, and colonic concentrations of propionate, butyrate and BCFA in the CON nursery group compared to the GH nursery group (*P* < 0.05; Table 8). Furthermore, within the CON nursery group, the PGH suckling group had the highest colonic acetate and propionate concentrations (*P* < 0.05).

**Table 8:** Metabolic profiles in gastrointestinal sections of piglets euthanized at post-weaning d 10 and d 38^1^

Nursery treatments	CON			GH			SEM^2^	*P*_suckling_	*P*_nursery_	*P*_suckling×nursery_
Suckling treatments	CON	GH	PGH	CON	GH	PGH				
Ileal content at d 10 post-weaning										
Acetate, µmol/g DM^3^	151	147	151	95	90	78	19	0.916	**0.001**	0.888
Propionate, µmol/g DM	3.01	3.06	3.76	2.38	3.23	2.86	0.48	0.442	0.266	0.531
Ammonia, mg/g DM	0.35	0.36	0.28	0.30	0.29	0.17	0.04	0.065	**0.032**	0.685
Caecal content at d 10 post-weaning										
Acetate, µmol/g DM	508^b^	760^a^	556^b^	632	674	707	46	**0.018**	0.113	**0.037**
Propionate, µmol/g DM	207^b*^	273^a*^	236^ab*^	188	171	183	14	0.244	** < 0.001**	**0.031**
Butyrate, µmol/g DM	103*	134*	120*	79	79	74	18	0.661	**0.011**	0.676
Valerate, µmol/g DM	9.42	10.0	7.33	7.12	9.42	8.09	1.28	0.290	0.500	0.505
Ammonia, mg/g DM	0.42^a*^	0.35^b*^	0.36^ab*^	0.38^a^	0.27^b^	0.32^ab^	0.03	**0.027**	**0.046**	0.720
Colonic content at d 10 post-weaning										
Acetate, µmol/g DM	367^b^	478^a^	494^a^	397^ab^	345^b^	497^a^	34	**0.010**	0.249	0.062
Propionate, µmol/g DM	127^b*^	173^b*^	200^a*^	122^b^	105^b^	149^a^	13	0.**004**	** < 0.001**	0.078
Butyrate, µmol/g DM	95*	93*	117*	63	53	66	7	0.079	** < 0.001**	0.400
Valerate, µmol/g DM	10	9.73	12	6.70	8.86	11	2.11	0.271	0.285	0.667
BCFA^4^, µmol/g DM	8.64*	9.16*	9.20*	4.25	5.56	6.47	1.33	0.580	**0.004**	0.828
Ammonia, mg/g DM	1.18	0.96	1.03	1.02	0.95	0.80	0.12	0.301	0.184	0.440
Ileal content at d 38 post-weaning										
Acetate, µmol/g DM	140	161	141	150	148	122	23	0.601	0.689	0.814
Ammonia, mg/g DM	0.12	0.08	0.08	0.09	0.09	0.10	0.02	0.210	0.663	0.197
Caecal content at d 38 post-weaning										
Acetate, µmol/g DM	880	907	877	932*	932*	902*	20	0.337	**0.046**	0.750
Propionate, µmol/g DM	306	348	339	319	332	329	19	0.342	0.813	0.721
Butyrate, µmol/g DM	191	183	181	185	178	179	21	0.921	0.796	0.993
Valerate, µmol/g DM	26	21	21	23	23	20	3	0.323	0.594	0.824
BCFA, µmol/g DM	7.82^a^	8.99^a^	6.41^b^	8.76^a^	8.07^a^	6.72^b^	0.75	**0.034**	0.859	0.469
Ammonia, mg/g DM	0.68	0.64	0.53	0.49	0.49	0.51	0.07	0.700	0.081	0.837
Colonic content at d 38 post-weaning										
Acetate, µmol/g DM	530	525	462	475	447	446	32	0.345	0.082	0.646
Propionate, µmol/g DM	215*	219*	197*	183	186	183	14	0.512	**0.042**	0.775
Butyrate, µmol/g DM	118	111	115	109	105	92	11	0.685	0.178	0.717
Valerate, µmol/g DM	15	16	13	12	14	11	2	0.103	0.084	0.721
BCFA, µmol/g DM	14^a^	12^ab^	9.71^b^	12^a^	9.06^ab^	9.71^b^	1.10	**0.020**	**0.029**	0.376
Ammonia, mg/g DM	1.18	1.37	1.07	1.05	1.00	1.04	0.10	0.494	0.056	0.320

^1^ Piglets were fed a control diet (CON nursery treatment) or a diet containing 28% (phase 1; d 0–14) or 13% (phase 2; d 14–39) grass pellets (GH nursery treatment) during the post-weaning period after being fed a control creep feed (CON), control creep feed and chopped grass hay (GH), or creep feed containing 28% grass pellets (PGH) during the suckling phase

^2^ Pooled standard error of the mean

^3 ^DM = dry matter

^4^ Sum of iso-butyrate and iso-valerate

^a,b^ Values within a row with different superscripts differ significantly based on suckling treatments at *P* < 0.05. Interaction effects were primarily marked when the significant difference in the overall means of suckling treatment was also observed

^*^ Indicates the significantly increased values within a row based on nursery treatments at *P* < 0.05

At d 38 post-weaning, caecal acetate concentration was higher in the GH nursery group than in the CON nursery group (932 vs. 888 µmol/g DM, *P* < 0.05), while colonic propionate and BCFA concentrations were higher in the CON nursery group (213 vs. 184, 12 vs. 10 µmol/g DM, respectively, *P* < 0.05; Table 8). Notably, PGH as suckling treatment led to a long-term effect, with lower levels of colonic BCFA compared to the other two suckling treatments (*P* < 0.05; (Tables 8 and 9).

**Table 9:** Alpha-diversity of caecal and colonic microbiota at weaning^1^

Items	CON	GH	PGH	SEM^2^	*P*_diet_
Caecal microbiota					
Shannon	5.20	4.80	5.43	0.43	0.355
Simpson	0.93	0.87	0.94	0.04	0.279
Chao1	404	412	454	47	0.530
Colonic microbiota					
Shannon	5.54	5.34	5.59	0.30	0.700
Simpson	0.94	0.93	0.93	0.02	0.719
Chao1	447	405	469	42	0.316

^1^ Piglets were fed one of three experimental diets: control creep feed (CON), control creep feed and chopped grass hay (GH), or creep feed containing 28% grass pellets (PGH)

^2 ^Pooled standard error of the mean

### Microbiota composition in cecum and mid-colon

The microbiota community in both cecum and colon showed no difference in α-diversity at the day prior to weaning as assessed by the Shannon, Simpson and Chao1 indices (*P* > 0.05). Post-weaning, the Chao1 index of the caecal microbiota was higher (*P* < 0.05) in the GH nursery group compared to the CON nursery group on d 10. The Shannon index was higher (*P* < 0.05) in the GH nursery group compared to the CON nursery group on d 38 (Table 10). In the colon, the Chao1 was higher (*P* < 0.05) in the GH nursery group compared to the CON nursery group on d 38. In the β-diversity analysis, significantly distinct microbiota communities among all groups were observed only on d 10 post-weaning, both in cecum (*P* = 0.003, *R* = 0.1951) and colon (*P* = 0.027, *R* = 0.1653, Fig. 3).

**Table 10:** Alpha-diversity of caecal and colonic microbiota on d 10 and d 38 post-weaning^1^

Nursery treatments	CON			GH			SEM^2^	*P*_suckling_	*P*_nursery_	*P*_suckling×nursery_
Suckling treatments	CON	GH	PGH	CON	GH	PGH				
Caecal microbiota on day 10 post-weaning										
Shannon	4.02	4.81	3.50	4.70	4.47	4.76	0.48	0.749	0.347	0.499
Simpson	0.78	0.88	0.69	0.88	0.75	0.83	0.07	0.759	0.643	0.372
Chao1	344	380	247	433*	486*	500*	25	0.267	** < 0.001**	0.074
Caecal microbiota on day 38 post-weaning										
Shannon	3.96	3.76	4.80	4.63*	5.96*	5.40*	0.59	0.397	**0.028**	0.335
Simpson	0.80	0.77	0.88	0.84	0.92	0.90	0.05	0.322	0.074	0.399
Chao1	390	372	430	375	593	486	47	0.324	0.131	0.225
Colonic microbiota on day 10 post-weaning										
Shannon	5.31	5.40	5.16	4.73	4.73	5.73	0.51	0.660	0.589	0.413
Simpson	0.92	0.93	0.92	0.83	0.80	0.92	0.05	0.671	0.175	0.553
Chao1	465	452	462	439	459	504	24	0.401	0.532	0.419
Colonic microbiota on day 38 post-weaning										
Shannon	4.99	5.40	5.48	5.65	6.24	5.59	0.215	0.415	0.095	0.600
Simpson	0.86	0.91	0.91	0.91	0.94	0.90	0.03	0.544	0.547	0.671
Chao1	528	552	549	627*	655*	610*	30	0.791	0.019	0.859

^1^ Piglets were fed a control diet (CON nursery treatment) or a diet containing 28% (phase 1; d 0–14) or 13% (phase 2; d 14–39) grass pellets (GH nursery treatment) during the post-weaning period after being fed a control creep feed (CON), control creep feed and chopped grass hay (GH), or creep feed containing 28% grass pellets (PGH) during the suckling phase

^2^ Pooled standard error of the mean

^*^ Indicates the significantly increased values within a row based on nursery treatments at *P* < 0.05

**Fig. 3:**
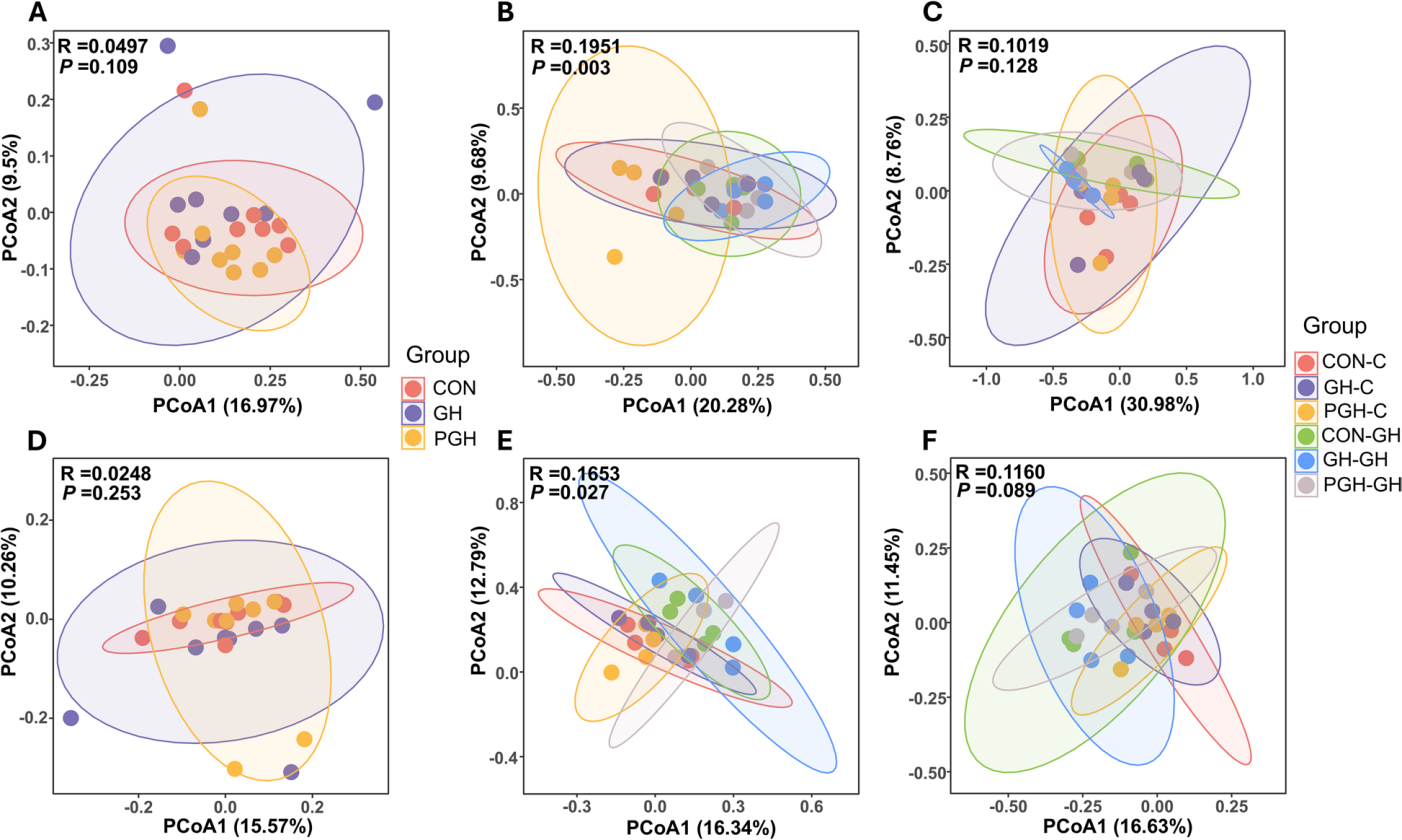
Beta-diversity of microbiota in cecum and mid-colon. Principal coordinates analysis (PCoA) of OTUs based on Unweighted UniFrac distances with ANOSIM test. **A–C** PCoA plots of cecal microbiota at weaning, day 10 and day 38 post-weaning. **D–F** PCoA plots of colonic microbiota at weaning, d 10 and d 38 post-weaning. CON = control group; GH = chopped grass hay group; PGH = pellet grass hay group. CON-C, GH-C and PGH-C represents piglets from CON, GH or PGH groups before weaning and followed by control nursery feed. CON-GH, GH-GH and PGH-GH represents piglets from CON, GH or PGH groups before weaning and followed by grass hay nursery feed

**Fig. 4:**
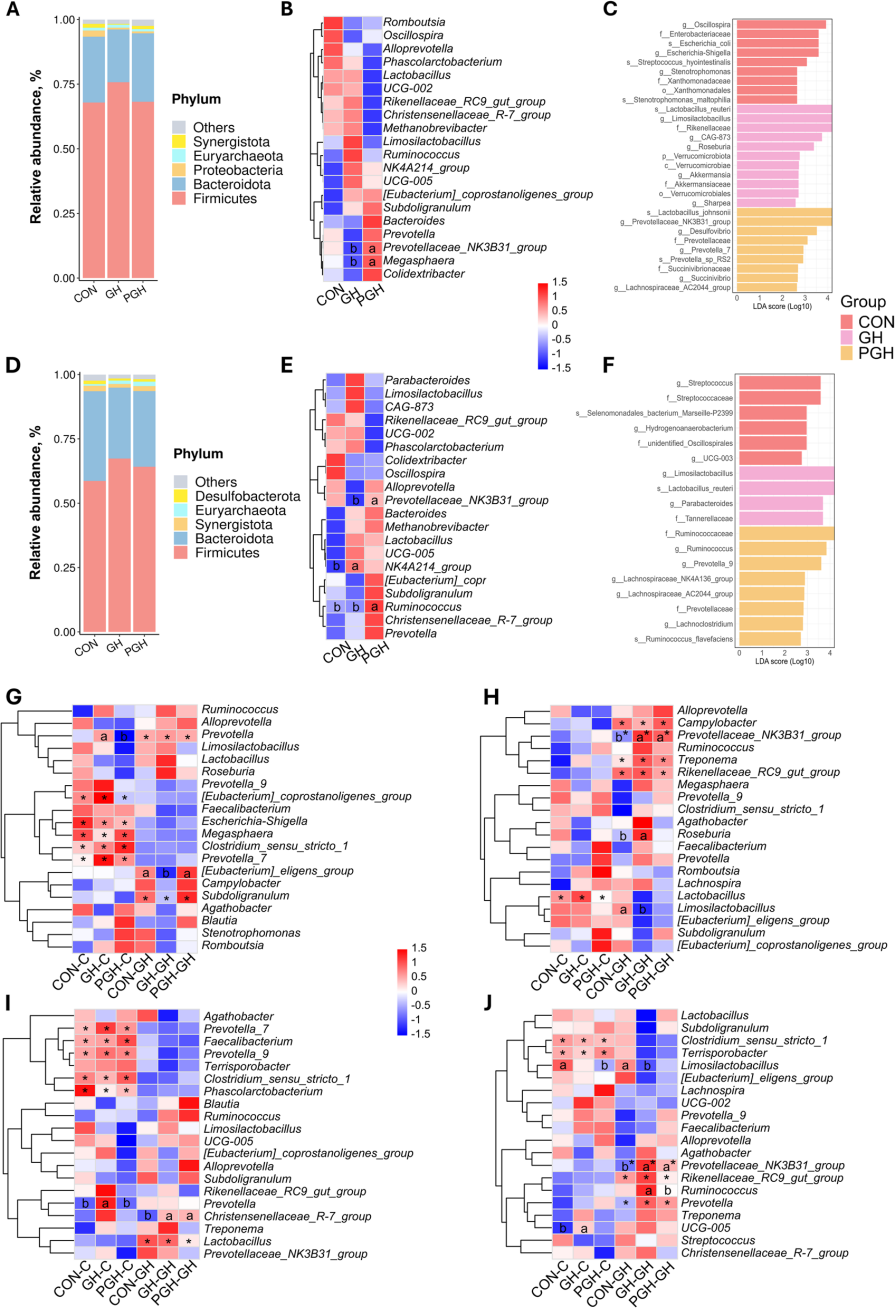
The microbiota composition and differential analysis in cecum and mid-colon across treatments and development stages. **A** Relative abundance of cecal microbiota on genus level at weaning. **B** Clustered heatmap of top 20 abundant genus in cecum based on log_2_-transformed data and Z-score normalization at weaning. **C** Histogram generated from linear discriminant analysis (LDA) effect size (LEfSe) analysis results for cecal microbiota at weaning. **D** Relative abundance of colonic microbiota on genus level at weaning. **E** Clustered heatmap of top 20 abundant genus in colon based on log_2_-processed and Z-score normalization. **F** Histogram generated from LEfSe results for colonic microbiota at weaning. Highlighting significant difference, taxa with difference at an LDA score greater than 2.5 were displayed in LEfSe histogram. **G **and** H** Clustered heatmap of top 20 abundant genus in cecum on day 10 and day 38 post-weaning. **I **and** J** Clustered heatmap of top 20 abundant genus in colon on d 10 and d 38 post-weaning. CON-C, GH-C and PGH-C represents piglets from CON, GH or PGH groups before weaning and followed by control nursery feed. CON-GH, GH-GH and PGH-GH represents piglets from CON, GH or PGH groups before weaning and followed by grass hay nursery feed. Kruskal–Wallis H test with Welch’s post-hoc test were applied in heatmap. ^a,b^Box with different superscripts differ significantly based on suckling treatments at *P* < 0.05; *Indicates a significant difference within a genus based on nursery treatments at *P* < 0.05

The microbiota structures were further analysed in cecum and mid-colon. Firmicutes (70.5%), Bacteroidetes (24.1%) and Proteobacteria (1.2%) were the three most abundant phyla in the cecum, while Firmicutes (63.3%), Bacteroidetes (30.6%) and Synergistota (1.8%) were predominant in the mid-colon at weaning (Fig. 4A and D). At genus level, *Prevotellaceae_NK3B31_group* and *Megasphaera* showed significantly higher abundance in the PGH suckling group compared to the GH suckling group within the top 20 abundant genera of cecum at weaning (*P* < 0.05, Fig. 4B). *Prevotellaceae_NK3B31_group* and *Ruminococcus* in the mid-colon were also more abundant in the PGH suckling group compared to other two groups (*P* < 0.05; Fig. 4E). LEfSe analysis revealed that the caecal microbiota in the CON suckling group compared to PGH and GH were rich in *Oscillospira, Escherichia-Shigella* abundance at the genus level and *Enterobaceriaceae* at the family level*.* The GH suckling group exhibited higher abundance in genera *Lactobacillus reuteri*, *Limosilactobacillus* and Rikenellaceae family in cecum, while the genera *Lactobacillus johnsonii* and *Prevotellaceae_NK3B31_group* were prominent in the PGH suckling group (Fig. 4C). In the colon,* Limosilactobacillus* and *Lactobacillus reuteri* remained notable in the GH suckling group, and Ruminococcaceae and Prevotellaceae families showed higher abundance in the PGH suckling group compared to the other two groups, as indicated by LEfSe analysis (Fig. 4F).

The top 20 abundant genera were clustered in a heatmap to illustrate differences post-weaning, and LEfSe analysis results for each group were also listed (Additional file 1: Fig. S1). At d 10 post-weaning and compared to the GH nursery group, *[Eubacterium] coprostanoligenes group, Prevotella 7, Escherichia shigella, Megasphaera, Clostridium sensu stricto 1* were more abundant in caecal microbiota of the CON nursery group (*P* < 0.05; Fig. 4G). By d 38, only *Lactobacillus* exhibited higher abundance in the CON nursery group (*P* < 0.05), whereas the *Prevotellaceae NK3B31 group* was more abundant in the GH nursery group in caecal microbiota (*P* < 0.05; Fig. 4H). Within the GH nursery group, both GH-GH and PGH-GH suckling treatment showed a higher abundance of *Prevotellaceae NK3B31 group* than the CON-GH suckling treatment (*P* < 0.05) both in cecum and colon on d 38 post-weaning (Fig. 4H and J). In the mid-colon, the CON nursery group showed higher abundances of *Prevotella 7, Prevotella 9, Faecalibacterium*, *Clostridium sensu stricto 1* and *Phascolarctobacterium* on d 10 post-weaning with *Clostridium sensu stricto 1* remaining elevated on d 38 (*P* < 0.05; Fig. 4I and J). In contrast, the GH nursery group showed a higher abundance of *Lactobacillus* on d 10 post-weaning, and *Prevotellaceae NK3B31 group* and *Prevotella* on d 38 post-weaning (*P* < 0.05; Fig. 4I and J).

## Discussion

We here demonstrate that introducing a fibrous diet item such as grass hay in the suckling phase, either separately or mixed in the creep feed, can influence the development of the GIT. It also can modify the fermentation and microbiota profile, with persisting effects into later life. Fibrous diets have been long recognized as beneficial for growing pigs and sows [22–24]. However, the variability in feed intake and unpredictable challenges associated with neonatal piglets have led to hesitation in exploring the fibre application for this age group.

Unlike our previous study, where the feed intake of chopped grass hay reached 54 g per piglet at weaning, the intake in the present study was limited to approximately 7 g per piglet (assuming equal intake among piglets in a litter) [15]. The shorter suckling duration (23 vs. 28 d) and the potential breed variation (Topigs Norsvin TN70 × Belgian Pietrain vs. Hypor Libra × Hypor Maxter) likely contributed to the difference in grass hay intake, as seen in other studies [25, 26]. In the previous study, a considerable increase in solid feed consumption was observed during the last week of lactation. Moreover, another study reported that piglets consumed only 5 g/piglet solid feed until d 20 of age, but this amount increased to 63 g/piglet in the following week [26]. This suggests that extending the suckling phase by one more week has a substantial impact on piglets’ overall appetite, highlighting the crucial role of suckling duration on grass hay consumption and its effects on GIT development. Particularly in light of the EU animal welfare legislation, the requirement for a longer suckling duration in practice may enhance the functional benefits of grass hay [27].

In the current study, despite the low grass hay intake, it is remarkable that substantial effects on intestinal development and microbial activity were found. The creep feed intake showed high variability between litters, with an approximate 70% coefficients of variation, as is commonly observed [6, 28, 29]. Growth performance during the suckling period is mainly determined by sow milk intake rather than creep feed intake, as shown in the current study [30]. Despite lower net energy consumption in the PGH fed piglets, this feed did not affect growth until weaning. Moreover, it may be due to the overestimation in the energy requirement of creep feed may in the modern farming practicing. In terms of dietary energy dilution, it seemed that the 28% inclusion rate of grass pellets (25.9% IDF) was more than the piglets could compensate for shortfall in energy intake. Interestingly their growth recovered and even surpassed the CON nursery groups when the grass pellet inclusion was reduced to 13% after d 14 post-weaning. Meanwhile, the optimized ECR was observed in PGH group throughout the study. In analogy, diets supplemented with 5% or 6% wheat bran enhanced growth performance in weaned piglets in contrast with a lack of effect of a higher inclusion rate of 14.9% in other studies [31–33]. Meanwhile, similar inclusion ratios of insoluble fibre, yet from different sources, yielded varying growth outcomes in weaned piglets [31]. Therefore, the optimal proportion of grass pellets in the nursery feed could be further optimized, considering the catch-up growth after an initial post-weaning growth retardation due to a high-fibre diet as shown in the present study. Considering the common challenge of highly variable creep feed intake, which was still observed in the current study, a further larger-scale experiment covering more piglet breeds across different regions may strengthen the findings.

In contrast to the grass pellet incorporated into the feed, the provision of chopped grass hay in a separate feeder increased the size of the GIT at weaning. This finding suggests that the physical form and the method of provision of chopped grass hay promoted this change. The large, coarse matter of insoluble fibre were reported to reduce digesta segregation (from mouth to ileocecal junction) in growing pigs [34], likely increasing bulk and retention time within specific sections of the GIT. A similar observation in finishing pigs, where those fed with coarse straw exhibited a longer median retention time throughout the intestinal tract compared to those fed fine straw [35]. This bulk, in turn, may enhance nutrient absorptive processes by activating mechanoreceptors (stretch activated neural cells), which promote digestive tract development [36]. This process also aligns with the observed long-term increase in tight junction protein expression in the colon as observed in this study. The lower post-weaning colon weight due to pre-weaning grass hay provision (independently of the nursery diet) may be the result of the sudden removal of grass hay as a physical stimulus, which is supported by the heavier colon when nursery piglets still received insoluble fibre through the pellet. Although both the ADFI and ADG were reduced in the GH nursery group compared to the CON nursery group, the FCR shows that the reduction in ADG is not fully explained by the reduction in ADFI. For instance, intestinal development requires a substantial amount of energy expenditure [37], which may explain the association between hay-induced intestine development and (temporary) overall growth reduction. This effect on colon development did not seem to be a measure of fermentative capacity, as colon weight effects often coincided with the inverse effect on SCFA production. Still, the LEfSe analysis revealed that taxa that were abundant in colon of piglets from PGH at weaning, such as *Ruminococcus*, *Prevotella*, *Prevotella 9* and *Prevotella* sp. RS2, became more prevalent in the colon of piglets from GH-GH on d 10 post-weaning. The management of GH-GH piglets, which initially had access to two feeders with free-choice feeding before weaning, transitioning to a single unfamiliar diet after weaning may also contributed to this intestinal growth stasis. Moreover, the abrupt shift of microbiota mentioned above in GH-GH may have intensified the weaning challenge [38, 39]. However, a better adaptation (similar abundant microbes) was seen in piglets that remained on feed that included grass pellets from pre- to post-weaning. The observed trend of an interactive effect in body weight of PGH-GH group may further suggest this adaptation mechanism besides its effect on luminal microbiota. Eventually, a long-term exposure to nursery feed containing the grass pellets, i.e., high insoluble fibre, resulted in an enlargement of the stomach and small and large intestines in piglets by the end of study. This is consistent with findings from other studies involving high- (insoluble) fibre diets [40–43].

The elevated expression of nutrient transporters and intestinal barrier proteins due to grass hay reflected the increased exposure to substrates as well as nutrient absorption efficiency within the intestinal lumen. Piglets that received the PGH diet during suckling had upregulated nutrient transporters, including *SGLT1*, *B0AT1* and *PepT1* in ileum, up to d 10 post-weaning, and piglets that received the GH nursery diet showed the same activation of these genes compared to the CON nursery diet by d 38 post-weaning. The results of transporters in ileum, together with the observed lower ammonia concentration in ileum at d 10 post-weaning, suggest that proximal intestinal segments of piglets fed a diet containing grass pellets at a young age became more capable of digesting dietary protein or producing monosaccharides [44–47]. However, additional data of on ileal microbiota and digestive enzyme activity may strengthen this conclusion. In the colon, the lower non-fibre carbohydrate level in GH nursery diets accompanied by lower concentrations of substrate availability, unsurprisingly resulted in lower expression levels of *FFAR2* and *MCT1* on d 38 post-weaning, consistent with previous studies linking lower expression levels to substrate availability [48–50]*.* Conversely, the higher SCFA levels in the colon of piglets from the PGH suckling group compared to the other two suckling treatments across both nursery treatments likely contributed to stimulation of the expression of *MCT1*.

Similar to findings from studies on (insoluble) fibre, fermentation primarily occurred in the cecum and proximal colon in pigs, as observed through the slaughter method [51–53]. A higher SCFA production was noted in the cecum of piglets supplemented with chopped GH on the day prior to weaning, whereas no such increase was shown in piglets fed a diet containing grass pellets during suckling. According to the data of caecal and colonic microbiota, this outcome was likely not due to a lack of carbohydrate fermentation capacity but rather the limited availability of fermentable substrates in PGH diets, including starch. Compared to the other two suckling treatments, piglets in the PGH group displayed a higher abundance of *Prevotellaceae NK3b31 group*, *Prevotella 7*, *Lachnospiraceae*, and *Lachnoclostridium* in the cecum before weaning. These abundant genera play crucial roles in degrading various types of dietary fibre in the diet [54–57], indicating that the inclusion of grass pellets in a diet induced more fibre fermentation in the cecum than separate provision of chopped grass hay. Because the voluntary intake of the separate grass hay was lower than anticipated, the fibre intake was higher through the inclusion of the grass pellet in the PGH group, which is a plausible explanation for the stronger effect on fermentation. Long-term feeding of grass hay pellet showed that the carbohydrate sources from the diet, exposed to the well-established taxa mentioned above in caecum, may be priorly fermented in caecum and absorbed in the longer colon. The results of SCFA distribution along GIT of piglets in GH nursery diet also match this hypothesis, along with an optimized ECR across the GH nursery group. Additionally, the higher SCFA levels in the proximal sections with lower pH environment can also downregulate bacterial urease activity, as reported in other studies [58–60]. Consequently, the resulting decrease in ammonia levels of digesta reduced the burden on the distal sections, as elevated ammonia concentrations can irreversibly deteriorate the intestinal barrier function [61].

It is, however, worth mentioning that the insoluble fibre content of grass hay is likely very unfermentable in young piglets especially when provided in coarse form. Therefore, its stimulating effect on fermentation is more likely due to passage rate kinetics rather than as substrate on its own. A similar observation was done in a piglet study comparing soluble with insoluble fibre where also the insoluble fibre had the largest effect on promoting fermentation [13]. The dominant caecal genera in piglets from the GH suckling group, such as *Limosilactobacillus* and *Lactobacillus_reuteri,* which are primarily utilizing glucose, lactose and maltose, coupled with higher SCFA levels, suggest that chopped grass hay effectively promoted fermentation of carbohydrate from the control creep feed or sow milk [62–64]. Additionally, the relatively lower caecal abundance of pathogens such as *Escherichia coli* and *Shigella* in the GH and PGH suckling groups compared to CON at weaning may also be attributed to the presence of SCFA substrates and acid-producing microbes [65–67]. Subsequently, a greater proportion of undigested sugars or starch from CON diets likely entered the colon, where they were fermented into higher concentrations of SCFA, facilitated by microbes such as *Streptococcus* and *Hydrogenoanaerobacterium* which were abundantly present [68, 69]*.* In contrast, in the PGH suckling group, the residual grass pellet content may have continued to support the growth of complex carbohydrate- or fibre-degrading genera in the colon, including *Prevotellaceae NK3b31 group*,* Ruminococcus*, and *Prevotella 9*. The similarity of abundant luminal microbes between the PGH suckling group and the GH nursery group might have facilitated the adaption to the diet transition around weaning, ultimately minimizing the negative impact on growth performance despite the 28% inclusion of grass pellets in the post-weaning diet and even showing improved performance switching to 13% inclusion of grass pellets.

Interestingly, after weaning, genera enriched in the caecum or colon of piglets fed the PGH diets during suckling, such as *Prevotella 7* and *Prevotella 9*, decreased when piglets were transitioned to GH nursery diets compared to CON nursery diets. This pattern suggests that pre-weaning abundance of these two genera in the PGH group may primarily be attributed to the components of sow milk, such as porcine milk oligosaccharides. Concurrently, the higher proportions of digestible carbohydrates in the CON nursery diets likely contributed to the abundance of *Clostridium sensu stricto 1* which has been reported as butyrate-forming pioneer in the early age of infants [70]. By the end of the study, with longer term of feeding GH nursery feed, the SCFA distribution in the proximal and distal segments exhibited a pattern similar with that observed in the GH and PGH suckling groups. Genera such as *Rikenellaceae RC9 gut group*, *Prevotellaceae NK3B31 group* and *Treponema* in the cecum, involved in fibre fermentation, may contribute to this pattern through prior exposure to other carbohydrates in the cecum during the digesta transit [54, 71, 72].

After all, a longer tracking on growth performance, such as following piglets until the slaughter phase, would provide a more comprehensive assessment of the results of larger GIT, enhanced intestinal barrier function, and altered SCFA distribution along with microbiota community shifts in large intestine induced by grass content.

## Conclusion

Providing chopped grass hay in a separate feeder next to a control creep feed to suckling piglets stimulated GIT development, hindgut fibre-degrading microbiota and shifted fermentation to more proximal parts of the intestinal tract, despite low intakes level during 23-day lactation. The GIT growth promotion was not seen when including 28% grass pellets in the creep feed, but that diet performed best in terms of ECR during the suckling and nursery phases.

After weaning, a 28% grass pellet inclusion in the feed reduced feed intake and weight gain for the first 2 weeks, yet piglets were shown to compensate and even increase weight gain which was mediated by an increase in feed intake once the grass pellet inclusion rate was reduced to 13%. Our study demonstrated that it is worth stepping away from the common approach of low-fibre and finely ground diets for young piglets. A consistent provision of a high-fibre diet for piglets is recommended if introduced during the suckling phase, given the beneficial effects on ECR and GIT health. Additionally, the inclusion rate of grass pellets right after weaning should be reconsidered and kept below 28%.

## Supplementary Information

Additional file 1: Table S1 The overview of primer sequences for target genes. Fig. S1 A–B Histogram generated from linear discriminant analysis (LDA) effect size (LEfSe) analysis results for cecal microbiota at d 10 and d 38 post-weaning. C–D Histogram generated from linear discriminant analysis (LDA) effect size (LEfSe) analysis results for colonic microbiota at d 10 post-weaning.

## Data Availability

The datasets used and/or analysed during the current study are available from the corresponding author on reasonable request.

## Abbreviations

ADF: Acid detergent fibre
ADL: Acid detergent lignin
ADG: Average daily gain
ADFI: Average daily feed intake
BW: Body weight
DE: Digestible energy
DM: Dry matter
ECR: Energy conversion ratio
FCR: Feed conversion ratio
GIT: Gastrointestinal tract
IDF: Insoluble dietary fibre
NDF: Neutral detergent fibre
NE: Net energy
OTU: Operational taxonomic unit
PCoA: Principal coordinates analysis
SCFA: Short-chain fatty acid
SID: Standard ileal digestibility

## References

[1] Lallès JP, Bosi P, Smidt H, Stokes CR. Nutritional management of gut health in pigs around weaning. Proc Nutr Soc. 2007;66(2):260–8. 10.1017/s0029665107005484. 17466106 10.1017/S0029665107005484

[2] Hervé J, Haurogné K, Buchet A, Bacou E, Mignot G, Allard M, et al. Pathogen exposure influences immune parameters around weaning in pigs reared in commercial farms. BMC Immunol. 2022;23:61. 10.1186/s12865-022-00534-z. 10.1186/s12865-022-00534-z PMC9737769 36496363

[3] Kick A, Tompkins M, Flowers W, Whisnant C, Almond G. Effects of stress associated with weaning on the adaptive immune system in pigs. J Anim Sci. 2012;90(2):649–56. 10.2527/jas.2010-3470. 21926316 10.2527/jas.2010-3470

[4] Colson V, Martin E, Orgeur P, Prunier A. Influence of housing and social changes on growth, behaviour and cortisol in piglets at weaning. Physiol Behav. 2012;107(1):59–64. 10.1016/j.physbeh.2012.06.001. 22691708 10.1016/j.physbeh.2012.06.001

[5] Amadori M, Razzuoli E, Nassuato C. Issues and possible intervention strategies relating to early weaning of piglets. CABI Reviews. 2012.p.1–15. 10.1079/pavsnnr20127046.

[6] Sulabo RC, Jacela JY, Tokach MD, Dritz SS, Goodband RD, DeRouchey JM, et al. Effects of lactation feed intake and creep feeding on sow and piglet performance. J Anim Sci. 2010;88(9):3145–53. 10.2527/jas.2009-2131. 20495122 10.2527/jas.2009-2131

[7] Van den Brand H, Wamsteeker D, Oostindjer M, van Enckevort LC, van der Poel AF, Kemp B, et al. Effects of pellet diameter during and after lactation on feed intake of piglets pre- and postweaning. J Anim Sci. 2014;92(9):4145–53. 10.2527/jas.2014-7408. 25185217 10.2527/jas.2014-7408

[8] Gresse R, Chaucheyras-Durand F, Fleury MA, Van de Wiele T, Forano E, Blanquet-Diot S. Gut microbiota dysbiosis in postweaning piglets: understanding the keys to health. Trends Microbiol. 2017;25(10):851–73. 10.1016/j.tim.2017.05.004. 28602521 10.1016/j.tim.2017.05.004

[9] Van Hees HMJ, Ballari SA, Dieste-Pérez L, Carpinetti BN, Janssens GPJ. Diet and stomach characteristics of feral piglets (Sus scrofa): Implications for farmed piglets. J Anim Physiol Anim Nutr. 2023;107(2):529–40. 10.1111/jpn.13726. 10.1111/jpn.13726 35603976

[10] Forbes JM. A personal view of how ruminant animals control their intake and choice of food: minimal total discomfort. Nutr Res Rev. 2007;20(2):132–46. 10.1017/s0954422407797834. 19079866 10.1017/S0954422407797834

[11] Raubenheimer D, Simpson SJ. Integrative models of nutrient balancing: application to insects and vertebrates. Nutr Res Rev. 1997;10(1):151–79. 10.1079/nrr19970009. 19094262 10.1079/NRR19970009

[12] Blaxter KL, Wilson RS. The assessment of a crop husbandry technique in terms of animal production. Anim Prod. 1963;5(1):27–42. 10.1017/S0003356100021486.

[13] Van Hees H, Davids M, Maes D, Millet S, Possemiers S, Hartog LA, et al. Dietary fibre enrichment of supplemental feed modulates the development of the intestinal tract in suckling piglets. J Anim Sci Biotechnol. 2019;10:83. 10.1186/s40104-019-0386-x. 10.1186/s40104-019-0386-x PMC6794736 31636904

[14] Van Hees HMJ, Chiers K, den Hartog LA, van Kempen TATG, Maes D, Millet S, et al. Supplementing oat hulls to the diet of suckling piglets altered their intestinal tract and colonic microbiota development. Anim Nutr. 2023;12:284–96. 10.1016/j.aninu.2022.10.002. 37013081 10.1016/j.aninu.2022.10.002 PMC10065989

[15] Yao R, Cools A, van Hees HMJ, Chiers K, Mebratu AT, Aluwé M, et al. Getting clues from nature: the impact of grass hay on suckling piglets’ gastrointestinal growth and colonic microbiota. Front cell infect microbiol. 2023;13:1341147. 10.3389/fcimb.2023.1341147. 38268791 10.3389/fcimb.2023.1341147 PMC10806113

[16] Slama J, Schedle K, Wurzer GK, Gierus M. Physicochemical properties to support fibre characterization in monogastric animal nutrition. J Sci Food Agric. 2019;99(8):3895–902. 10.1002/jsfa.9612. 30684273 10.1002/jsfa.9612 PMC6767034

[17] Williams S. Official methods of analysis of the Association of Official Analytical Chemists. Association of Official Analytical Chemists, Inc.; 1984.

[18] EC. Commission Regulation (EC) No 152/2009 of 27 January 2009 laying down the methods of sampling and analysis for the official control of feed. Official Journal of the European Union L. 2009;54:1-130

[19] Goering HK,Van Soest PJ. Forage fiber analyses (apparatus, reagents, procedures, and some applications). US Agricultural Research Service; 1970.

[20] Gadeyne F, Ruyck K, Ranst G, De Neve N, Vlaeminck B, Fievez V. Effect of changes in lipid classes during wilting and ensiling of red clover using two silage additives on in vitro ruminal biohydrogenation. J Agric Sci. 2016;1:1–14. 10.1017/S0021859615001203.

[21] Chaney AL, Marbach EP. Modified reagents for determination of urea and ammonia. Clin Chem. 1962;8(2):130–2. 13878063

[22] Jin L, Reynolds L, Redmer D, Caton J, Crenshaw J. Effects of dietary fiber on intestinal growth, cell proliferation, and morphology in growing pigs. J Anim Sci. 1994;72(9):2270–8. 10.2527/1994.7292270x. 7528192 10.2527/1994.7292270x

[23] McGlone J, Fullwood S. Behavior, reproduction, and immunity of crated pregnant gilts: effects of high dietary fiber and rearing environment. J Anim Sci. 2001;79(6):1466–74. 10.2527/2001.7961466x. 11424683 10.2527/2001.7961466x

[24] Gao Q, Liu Z, Li K, Bai G, Liu L, Zhong R, et al. Time-course effects of different fiber-rich ingredients on energy values, microbiota composition and SCFA profile in growing pigs. Anim Nutr. 2023;12:263–75. 10.1016/j.aninu.2022.10.003. 36712404 10.1016/j.aninu.2022.10.003 PMC9868344

[25] Baumung R, Lercher G, Willam A, Sölkner J. Feed Intake Behaviour of different pig breeds during performance testing on station. Archiv fur Tierzucht. 2006;49:77-88. 10.5194/aab-49-77-2006.

[26] Pajor EA, Fraser D, Kramer DL. Consumption of solid food by suckling pigs: individual variation and relation to weight gain. Appl Anim Behav Sci. 1991;32(2):139–55. 10.1016/S0168-1591(05)80038-3.

[27] Council of the European union. Council Directive 2008/120/EC of 18 December 2008 laying down minimum standards for the protection of pigs. 2008; 14.12.2019: Available from: http://data.europa.eu/eli/dir/2008/120/oj.

[28] Bruininx EM, Binnendijk GP, van der Peet-Schwering CM, Schrama JW, den Hartog LA, Everts H, et al. Effect of creep feed consumption on individual feed intake characteristics and performance of group-housed weanling pigs. J Anim Sci. 2002;80(6):1413–8. 10.2527/2002.8061413x. 12078720 10.2527/2002.8061413x

[29] Middelkoop A, Choudhury R, Gerrits W, Kemp B, Kleerebezem M, Bolhuis J. Effects of creep feed provision on behavior and performance of piglets around weaning. Front Vet Sci. 2020;7:520035. 10.3389/fvets.2020.520035. 10.3389/fvets.2020.520035 PMC7689248 33282925

[30] Pluske JR, Kim JC, Hansen CF, Mullan BP, Payne HG, Hampson DJ, et al. Piglet growth before and after weaning in relation to a qualitative estimate of solid (creep) feed intake during lactation: a pilot study. Arch Anim Nutr. 2007;61(6):469–80. 10.1080/17450390701664249. 18069618 10.1080/17450390701664249

[31] Yu C, Zhang S, Yang Q, Peng Q, Zhu J, Zeng X, et al. Effect of high fibre diets formulated with different fibrous ingredients on performance, nutrient digestibility and faecal microbiota of weaned piglets. Arch Anim Nutr. 2016;70(4):263–77. 10.1080/1745039X.2016.1183364. 27216554 10.1080/1745039X.2016.1183364

[32] Zhao J, Liu P, Wu Y, Guo P, Liu L, Ma N, et al. Dietary fiber increases butyrate-producing bacteria and improves the growth performance of weaned piglets. J Agric Food Chem. 2018;66(30):7995–8004. 10.1021/acs.jafc.8b02545. 29986139 10.1021/acs.jafc.8b02545

[33] Shang Q, Ma X, Liu H, Liu S, Piao X. Effect of fibre sources on performance, serum parameters, intestinal morphology, digestive enzyme activities and microbiota in weaned pigs. Arch Anim Nutr. 2020;74(2):121–37. 10.1080/1745039X.2019.1684148. 31821028 10.1080/1745039X.2019.1684148

[34] Dorado-Montenegro S, Lammers-Jannink K, Gerrits W, de Vries S. Insoluble fibers affect digesta transit behavior in the upper gastrointestinal tract of growing pigs, regardless of particle size. J Anim Sci. 2023;101. 10.1093/jas/skad299. 10.1093/jas/skad299 PMC10651184 37665959

[35] Lannuzel C, Veersma RJ, Wever N, van Erven G, Kabel MA, Gerrits WJJ, et al. Particle size of insoluble fibres and gelation of soluble fibres influence digesta passage rate throughout the gastrointestinal tract of finishing pigs. animal. 2024;18(6):101175. 10.1016/j.animal.2024.101175. 10.1016/j.animal.2024.101175 38772078

[36] Furness JB. Types of neurons in the enteric nervous system. J Auton Nerv Syst. 2000;81(1–3):87–96. 10.1016/s0165-1838(00)00127-2. 10869706 10.1016/s0165-1838(00)00127-2

[37] Stojanović O, Altirriba J, Rigo D, Spiljar M, Evrard E, Roska B, et al. Dietary excess regulates absorption and surface of gut epithelium through intestinal PPARα. Nat Commun. 2021;12:7031. 10.1038/s41467-021-27133-7. 34857752 10.1038/s41467-021-27133-7 PMC8639731

[38] Fouhse JM, Zijlstra RT, Willing BP. The role of gut microbiota in the health and disease of pigs. Anim Front. 2016;6(3):30–6. 10.2527/af.2016-0031.

[39] Guevarra RB, Hong SH, Cho JH, Kim BR, Shin J, Lee JH, et al. The dynamics of the piglet gut microbiome during the weaning transition in association with health and nutrition. J Anim Sci Biotechnol. 2018;9:54. 10.1186/s40104-018-0269-6. 30069307 10.1186/s40104-018-0269-6 PMC6065057

[40] Jørgensen H, Zhao X-Q, Eggum B. The influence of dietary fibre and environmental temperature on the development of the gastrointestinal tract, digestibility, degree of fermentation in the hind-gut and energy metabolism in pigs. Br J Nutr. 1996;75:365–78. 10.1079/BJN19960140. 8785211 10.1079/bjn19960140

[41] Len NT, Hong TTT, Ogle B, Lindberg JE. Comparison of total tract digestibility, development of visceral organs and digestive tract of Mong cai and Yorkshire × Landrace piglets fed diets with different fibre sources. J Anim Physiol Anim Nutr. 2009;93(2):181–91. 10.1111/j.1439-0396.2007.00804.x. 10.1111/j.1439-0396.2007.00804.x 19320931

[42] Priester M, Visscher C, Fels M, Dusel G. Influence of dietary fiber on the development of the gastrointestinal tract and the performance of gilts. Sustainability. 2020;12(12):4961. 10.3390/su12124961.

[43] Ivarsson E, Liu HY, Dicksved J, Roos S, Lindberg JE. Impact of chicory inclusion in a cereal-based diet on digestibility, organ size and faecal microbiota in growing pigs. Animal. 2012;6(7):1077–85. 10.1017/S1751731111002709. 23031467 10.1017/S1751731111002709

[44] Jean C, Rome S, Mathe V, Huneau J-F, Aattouri N, Fromentin G, et al. Metabolic evidence for adaptation to a high protein diet in rats. J Nutr. 2001;131(1):91–8. 10.1093/jn/131.1.91. 11208943 10.1093/jn/131.1.91

[45] Broer S. Amino acid transport across mammalian intestinal and renal epithelia. Physiol Rev. 2008;88(1):249–86. 10.1152/physrev.00018.2006. 18195088 10.1152/physrev.00018.2006

[46] Zhang S, Qiao S, Ren M, Zeng X, Ma X, Wu Z, et al. Supplementation with branched-chain amino acids to a low-protein diet regulates intestinal expression of amino acid and peptide transporters in weanling pigs. Amino Acids. 2013;45(5):1191–205. 10.1007/s00726-013-1577-y. 23990159 10.1007/s00726-013-1577-y

[47] Gorboulev V, Schürmann A, Vallon V, Kipp H, Jaschke A, Klessen D, et al. Na^+^-D-glucose Cotransporter SGLT1 is pivotal for intestinal glucose absorption and glucose-dependent incretin secretion. Diabetes. 2011;61(1):187–96. 10.2337/db11-1029. 22124465 10.2337/db11-1029 PMC3237647

[48] Shackley M, Ma Y, Tate EW, Brown AJH, Frost G, Hanyaloglu AC. Short chain fatty acids enhance expression and activity of the umami taste receptor in enteroendocrine cells via a Gα_i/o_ pathway. Front Nutr. 2020;7:568991. 10.3389/fnut.2020.568991. 10.3389/fnut.2020.568991 PMC7658341 33195366

[49] Frost G, Cai Z, Raven M, Otway DT, Mushtaq R, Johnston JD. Effect of short chain fatty acids on the expression of free fatty acid receptor 2 (Ffar2), Ffar3 and early-stage adipogenesis. Nutr diabetes. 2014;4(8):e128–e128. 10.1038/nutd.2014.25. 25089883 10.1038/nutd.2014.25 PMC4151174

[50] Cuff MA, Lambert DW, Shirazi-Beechey SP. Substrate-induced regulation of the human colonic monocarboxylate transporter, MCT1. Physiol J. 2002;539(Pt 2):361–71. 10.1113/jphysiol.2001.014241. 10.1113/jphysiol.2001.014241 PMC2290148 11882670

[51] Jha R, Berrocoso JFD. Dietary fiber and protein fermentation in the intestine of swine and their interactive effects on gut health and on the environment: A review. Anim Feed Sci Technol. 2016;212:18–26. 10.1016/j.anifeedsci.2015.12.002.

[52] Glitsø LV, Brunsgaard G, Højsgaard S, Sandström B, Bach Knudsen KE. Intestinal degradation in pigs of rye dietary fibre with different structural characteristics. Br J Nutr. 1998;80(5):457–68. 9924268

[53] Serena A, Jørgensen H, Bach Knudsen KE. Digestion of carbohydrates and utilization of energy in sows fed diets with contrasting levels and physicochemical properties of dietary fiber. J Anim Sci. 2008;86(9):2208–16. 10.2527/jas.2006-060. 18310490 10.2527/jas.2006-060

[54] Qiu Q, Zhu Y, Qiu X, Gao C, Wang J, Wang H, et al. Dynamic variations in fecal bacterial community and fermentation profile of Holstein steers in response to three stepwise density diets. Animals. 2019;9(8):560. 10.3390/ani9080560. 31443265 10.3390/ani9080560 PMC6719243

[55] Precup G, Vodnar DC. Gut *Prevotella* as a possible biomarker of diet and its eubiotic versus dysbiotic roles: a comprehensive literature review. Br J Nutr. 2019;122(2):131–40. 10.1017/s0007114519000680. 30924428 10.1017/S0007114519000680

[56] Ćesić D, LugovićMihić L, Ozretić P, Lojkić I, Buljan M, Šitum M, et al. Association of gut lachnospiraceae and chronic spontaneous urticaria. Life. 2023;13(6):1280. 10.3390/life13061280. 37374063 10.3390/life13061280 PMC10301119

[57] Wu YT, Shen SJ, Liao KF, Huang CY. Dietary plant and animal protein sources oppositely modulate fecal *Bilophila* and *Lachnoclostridium* in vegetarians and omnivores. Microbiol Spectr. 2022;10(2):e0204721. 10.1128/spectrum.02047-21. 35285706 10.1128/spectrum.02047-21 PMC9045121

[58] Jakhar D, Sarin SK, Kaur S. Gut microbiota and dynamics of ammonia metabolism in liver disease. npj Gut Liver. 2024;1:11. 10.1038/s44355-024-00011-x.

[59] Vince AJ, Burridge SM. Ammonia production by intestinal bacteria: the effects of lactose, lactulose and glucose. J Med Microbiol. 1980;13(2):177–91. 10.1099/00222615-13-2-177. 7381915 10.1099/00222615-13-2-177

[60] Zhu R, Liu L, Zhang G, Dong J, Ren Z, Li Z. The pathogenesis of gut microbiota in hepatic encephalopathy by the gut-liver-brain axis. Biosci Rep. 2023;43(6):BSR20222524. 10.1042/bsr20222524. 10.1042/BSR20222524 PMC10272964 37279097

[61] Wood CM, Liew HJ, De Boeck G, Hoogenboom JL, Anderson WG. Nitrogen handling in the elasmobranch gut: a role for microbial urease. J Exp Biol. 2019;222(3):jeb194787. 30530835 10.1242/jeb.194787

[62] Rodríguez-Sojo MJ, Ruiz-Malagón AJ, Rodríguez-Cabezas ME, Gálvez J, Rodríguez-Nogales A. *Limosilactobacillus fermentum* CECT5716: Mechanisms and therapeutic insights. Nutrients. 2021;13(3):1016. 10.3390/nu13031016. 10.3390/nu13031016 PMC8003974 33801082

[63] Zhao X, Gänzle MG. Genetic and phenotypic analysis of carbohydrate metabolism and transport in Lactobacillus reuteri. Int J Food Microbiol. 2018;272:12–21. 10.1016/j.ijfoodmicro.2018.02.021. 29505955 10.1016/j.ijfoodmicro.2018.02.021

[64] Wei X, Ouyang K, Long T, Liu Z, Li Y, Qiu Q. Dynamic variations in rumen fermentation characteristics and bacterial community composition during in vitro fermentation. Fermentation. 2022;8(6):276. 10.3390/fermentation8060276.

[65] Reid G, Burton J. Use of *Lactobacillus* to prevent infection by pathogenic bacteria. Microb Infect. 2002;4(3):319–24. 10.1016/S1286-4579(02)01544-7. 10.1016/s1286-4579(02)01544-7 11909742

[66] Nakayama SI, Watanabe H. Involvement of cpxA, a sensor of a two-component regulatory system, in the pH-dependent regulation of expression of Shigella sonnei virF gene. J Bacteriol. 1995;177(17):5062–9. 10.1128/jb.177.17.5062-5069.1995. 7665485 10.1128/jb.177.17.5062-5069.1995 PMC177285

[67] Ogawa M, Shimizu K, Nomoto K, Tanaka R, Hamabata T, Yamasaki S, et al. Inhibition of in vitro growth of Shiga toxin-producing *Escherichia coli* O157:H7 by probiotic *Lactobacillus* strains due to production of lactic acid. Int J Food Microbiol. 2001;68(1):135–40. 10.1016/S0168-1605(01)00465-2. 11545213 10.1016/s0168-1605(01)00465-2

[68] Jeong H, Lim YW, Yi H, Sekiguchi Y, Kamagata Y, Chun J. Anaerosporobacter mobilis gen. nov., sp. Nov., isolated from forest soil. Int J Syst Evol Microbiol. 2007;57(8):1784–7. 10.1099/ijs.0.63283-0. 17684257 10.1099/ijs.0.63283-0

[69] Van den Bogert Bv, Erkus O, Boekhorst J, Goffau Md, Smid EJ, Zoetendal EG, et al. Diversity of human small intestinal *Streptococcus* and *Veillonella* populations. FEMS Microbiol Ecol. 2013;85(2):376-388. 10.1111/1574-6941.12127. 10.1111/1574-6941.12127 23614882

[70] Appert O, Garcia AR, Frei R, Roduit C, Constancias F, Neuzil-Bunesova V, et al. Initial butyrate producers during infant gut microbiota development are endospore formers. Environ Microbiol. 2020;22(9):3909–21. 10.1111/1462-2920.15167. 32686173 10.1111/1462-2920.15167

[71] Graf J. The Family Rikenellaceae. In: Rosenberg E, DeLong EF, Lory S, Stackebrandt E, Thompson F, editors. The Prokaryotes: Other Major Lineages of Bacteria and The Archaea. Berlin, Heidelberg: Springer; 2014:857–859. 10.1007/978-3-642-38954-2_134.

[72] Ziołecki A. Isolation and characterization of large treponemes from the bovine rumen. Appl Environ Microbiol. 1979;37(1):131–5. 10.1128/aem.37.1.131-135.1979. 760632 10.1128/aem.37.1.131-135.1979 PMC243412

