# Supplementary material for: Grass hay mixed-in creep feed or separately-fed differentially affects digestive development in pre- and post-weaning piglets
Source: J Anim Sci Biotechnol. 2025 Jul 1;16:92. doi: 10.1186/s40104-025-01227-4 (PMC12211298; doi:10.1186/s40104-025-01227-4)
Supplement: Supplementary file 1 — Additional file 1: Table S1 The overview of primer sequences for target genes. Fig. S1 A–B Histogram generated from linear discriminant analysis (LDA) effect size (LEfSe) analysis results for cecal microbiota at d 10 and d 38 post-weaning. C–D Histogram generated from linear discriminant analysis (LDA) effect size (LEfSe) analysis results for colonic microbiota at d 10 post-weaning. [file 40104_2025_1227_MOESM1_ESM.docx]

**Table S1** The overview of primer sequences for target genes

| Genes | Sequence (5' -> 3') | Amplicon size, bp |
| --- | --- | --- |
| *SLC5A1* | F: GCCAGTAATATCGGAAGCGGGCA  R: ACAGCCAGCCCAGAACAACCA | 121 |
| *SLC6A19* | F: TCCAGACCTGCGACATGAACTCC  R: AAGAAGAGCACCGACCAGAGCG | 124 |
| *SLC15A1* | F: GTTCCTGCTGCTCTCATGGCTGT  R: GCAAACCCGATGCACTTGACGAC | 119 |
| *TJP1* | F: AGTCAACCCACCAAACCCACCA  R: TTGCCATCTCTTGCTGCCAAACT | 131 |
| *CLDN3* | F: CGGCAGCAGCATTATCACAGCG  R: AGCGCCAGCAGAGAGTCGTACA | 111 |
| *FFAR2* | F: ATCGTCTGTGCCCTCATGGGTT  R: ACAGCTTGTACTGCACGGGGAAA | 121 |
| *SLC16A1* | F: GGCCACCACTTTTAGGTCGTCTCA  R: CGGTAGTTGATGCCCATGCCGA | 124 |

*SLC5A1* = *SGLT1,*  sodium/glucose cotransporter 1; *SLC6A19 =* *B0AT1*, Sodium-dependent neutral amino acid transporter B(0)AT1; *SLC15A1 = PepT1*, Peptide transporter 1; *TJP1 = ZO-1*, Tight junction protein ZO-1; *CLDN3 =* Claudin 3; *FFAR2* = Free fatty acid receptor 2; *SLC16A1 = MCT1*, Monocarboxylate transporter 1


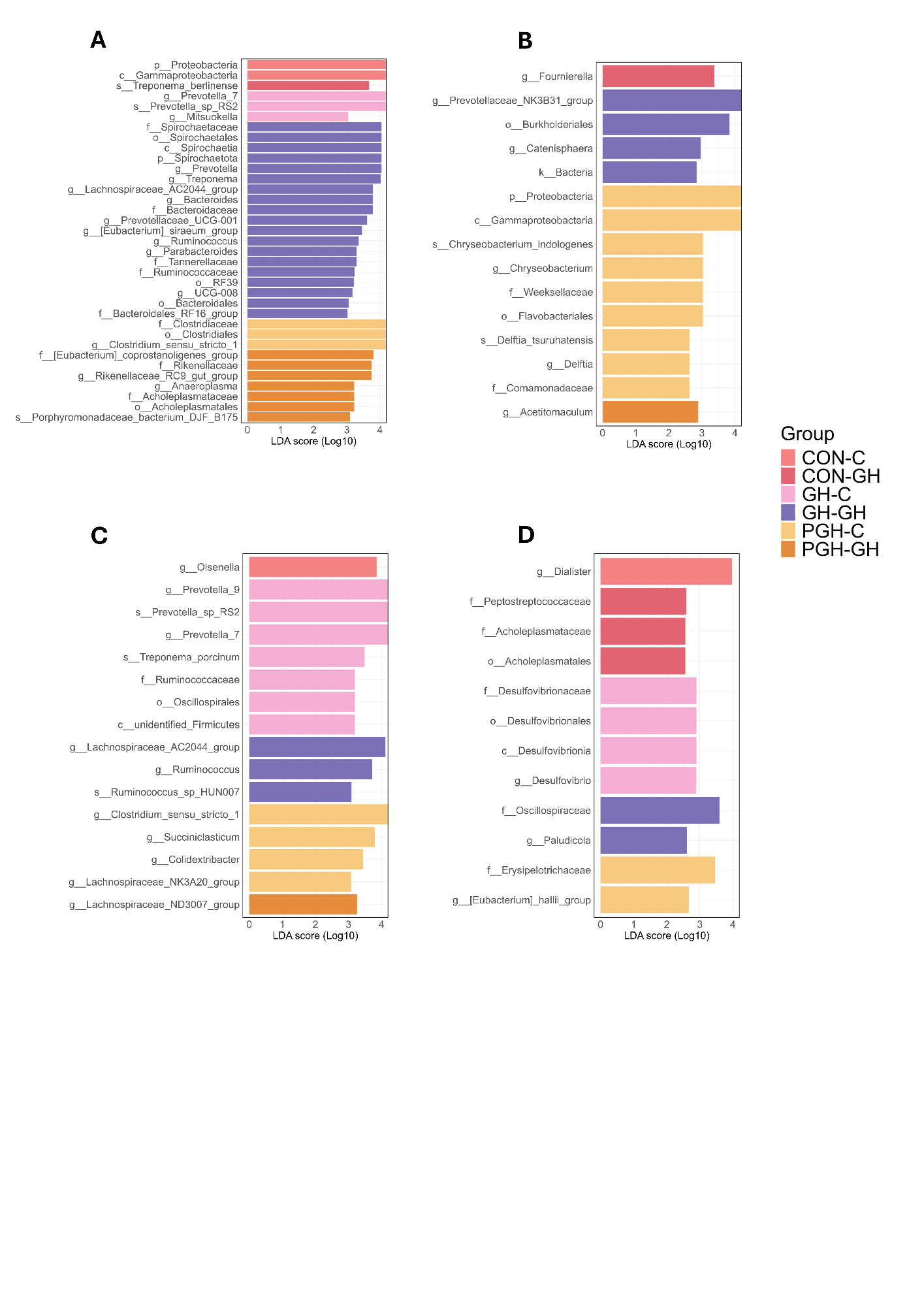


**Figure S1.** **(A-B)** Histogram generated from linear discriminant analysis (LDA) effect size (LEfSe) analysis results for cecal microbiota at d 10 and d 38 post-weaning. **(C-D)** Histogram generated from linear discriminant analysis (LDA) effect size (LEfSe) analysis results for colonic microbiota at d10 post-weaning. Highlighting significant difference, taxa with difference at an LDA score greater than 2.5 were displayed in LEfSe histogram. CON-C, GH-C and PGH-C represents piglets from CON, GH or PGH groups before weaning and followed by control nursery feed. CON-GH, GH-GH and PGH-GH represents piglets from CON, GH or PGH groups before weaning and followed by grass hay nursery feed.
